# Abstracts from the World Congress of Cardiology/Oriental Congress of Cardiology 2024

**DOI:** 10.5334/gh.1362

**Published:** 2024-11-18

**Authors:** The Editorial Team (On Behalf Of The World Heart Federation)

**Affiliations:** 1 On behalf of the World Heart Federation, Switzerland

**Keywords:** Cardiology, Global Health, Heart Health, Cardiovascular Disease, Conference

## Abstract

These are the top 30 abstracts from the combined 18th Oriental Congress of Cardiology together with the World Congress of Cardiology, held in June 2024. From 1950 to today, the World Heart Federation’s World Congress of Cardiology (WCC) has been a key event on the cardiovascular calendar, offering a global perspective on cardiovascular health and bringing together thousands of cardiology professionals from all over the world with one common goal: to reduce the global burden of cardiovascular disease and help people live longer, healthier lives.

Abstract ID: 380

Track: Atrial fibrillation

## Efficacy and Safety of Apixaban for Stroke Prevention in Atrial Fibrillation: A Systematic Review

Fernanda Santos da Anunciação^1^, Júlia Jamile Barbosa Palhares^2^, Rafaela Schelbauer^3^, Viviane Virgínia Araújo de Oliveira^4^, João Pedro do Valle Varela^4^, Sabrina Jorge Rodrigues^4^

(1) Universidade Estadual de Feira de Santana (UEFS); (2) Universidade de Franca (Unifran); (3) Universidade Estadual do Oeste do Paraná campus Cascavel; (4) Faculdade Metropolitana São Carlos

**Background & Objective:** A recognized risk factor for the incidence of strokes is an arrhythmia known as atrial fibrillation. It may manifest as either a clinical or subclinical condition. Understanding this condition’s classification and its respective treatment is highly studied. One of the resources used for this purpose is the CHADS(2), CHADS(2)DS(2)VASc, and HAS-BLED scales, which are utilized to predict the risk of stroke and bleeding in patients with atrial fibrillation during clinical trials. Additionally, identifying the treatment for stroke prevention due to atrial fibrillation involves investigating the use of apixaban, vitamin K antagonist (VKA) therapy, aspirin, and catheter ablation. The medication “Apixaban” is an inhibitor of factor Xa, with a 50% bioavailability and 25% renal excretion, effectively preventing venous thromboembolism (1,2,3,4). This study aims to evaluate whether apixaban is a more efficient method to prevent stroke in patients with atrial fibrillation compared to other therapies.

**Methodology:** This study consists of a systematic review with a mixed method to evaluate the efficacy and safety of apixaban in preventing stroke in patients diagnosed with atrial fibrillation (AF). The search strategy consists of exploring the databases PubMed, Embase, UpToDate, and Scopus, using the following search descriptors: “apixaban”, “atrial fibrillation”, “stroke prevention” and “clinical trial”. The inclusion criteria exclusively cover randomized controlled trials (RCTs) published between 2018 and 2025 that compared apixaban, aspirin, and vitamin K antagonists in patients diagnosed with AF. Four reviewers carried out the study selection independently, screening the titles and abstracts of articles identified in the initial search phase. Subsequently, the selected articles will be subjected to a full analysis to determine their eligibility according to the previously established inclusion and exclusion criteria. The data to be extracted comprises the study characteristics (author, year, study design), participant characteristics (age, sex, comorbidities), interventions, efficacy outcomes (stroke rate) and safety (bleeding occurrence).

**Results:** Four double-blind randomized clinical trials were employed in this review. Regarding stroke prevention, three studies indicate apixaban’s superiority over aspirin or vitamin K antagonists. One study focusing on patients undergoing ablation highlights apixaban’s non-inferiority to vitamin K antagonist therapy and underscores its safety. Concerning major bleeding risk, two studies associate apixaban, with one showing significant differences compared to aspirin. Contrary to this, the ablation study reveals no significant differences. Another study, compared to warfarin, emphasizes consistent major bleeding reduction with apixaban. Importantly, no significant mortality differences were observed between groups. Additionally, one study indicates a consistent reduction in net clinical events with apixaban across all age groups, suggesting benefits beyond stroke prevention.

**Conclusion:** In summary, the review indicates that apixaban exhibits superior efficacy in preventing strokes compared to aspirin and vitamin K antagonists in patients with atrial fibrillation. Despite concerns regarding the risk of major bleeding, the consistent reduction in net clinical events across all age groups underscores the potential benefits of apixaban beyond stroke prevention. Further research is required to validate these findings and optimize stroke prevention strategies in atrial fibrillation.

Abstract ID: 1187

Track: Coronary artery disease

## Secular Trends of Atrial Fibrillation as a Contributing Cause of Death in Chile

Ortiz M^1^, Morris R^1^, Asenjo R^1^, Cereceda M^1^.

1) Cardiovascular Department, Clinical Hospital, University of Chile

**Introduction:** There is little data on the impact and trends of atrial fibrillation (AF) on death (D) in the developing world.

**Objective:** To determine secular trends in age and gender specific D rates attributable to AF from 2000 to 2020 in Chile, a country with a life expectancy similar to that of the developed countries.

**Methods:** Data were based on D certificates and were obtained from Statistic National Institute, an agency of chilean government that captures health outcomes for the entire country. AF-related D are those for which the contributing causes of D listed by a physician is classified as code I 48 according to International Classification of Disease, Tenth Revision (Table 1).

**Table 1:** Death rates attributable to atrial fibrillation from 2000 to 2020 in Chile.


YEAR	00	02	04	06	08	10	12	14	16	18	20

Total*	2.0	2.7	3.4	4.1	4.6	5.5	6.2	6.0	5.5	4.8	5.0

**Gender**											

Male**	1.8	2.3	2.7	3.7	4.0	4.6	5.1	4.8	4.6	4.3	4.3

Female***	2.1	3.1	4.1	4.6	5.4	6.3	7.2	7.1	6.5	5.4	5.6

**Age (yrs)**											

40–59*	0.3	0.5	0.5	0.5	0.6	0.5	0.6	0.5	0.5	0.4	0.5

60–79**	9.4	11.1	13.8	16	15	15.4	15.8	13.5	12.2	10.1	9.7

≥80^+++^	60.6	90.5	118.8	148.7	150	194	172.3	166.6	145.8	123.8	139


**Note:** 95% CI(X̅): *4.5(3.7–5.3), **3.8(3.2–4.5), ***5.2(4.3–6.2), ^+^0.5(0.4–0.5), ^++^12.9(11.4–14.4), ^+++^137.3(111.9–162.6).

**Results:** Death rates per 100.000 population.

**Conclusion:** 1) This study represents the first evaluation of AF attributable D in Chile for 2 decades 2) Our data suggest that from 2000 to 2012 there was a 130% increase in the age-specific D rate attributable to AF in Chile and from 2014 onwards a decreasing trend was observed with a slight rebound in 2020. 3) This results show a high impact of AF on mortality in the developing world. The decrease in mortality rate in recent year suggest an improvement in the management of this arrhythmia and its comorbidities.

Abstract ID: 236

Track: Coronary artery disease

## Computed tomography angiography or invasive coronary angiography in patients with low-to-intermediate risk acute coronary syndrome – a single-center study

Dmitry Duplyakov^1^, Karina Kuznetsova^1^, Elena Adonina^1^

(1) Samara regional cardio center

**Introduction:** Emergency department (ED) attendances with chest pain requiring assessment for acute coronary syndrome (ACS) are a major global health issue. Due to the consequences of inadvertent discharge of a patient with missed ACS and the limitations of initial clinical assessment, most patients with suspected ACS will require diagnostic investigation and a short hospital admission or a period of observation in the ED. Coronary computed tomography angiography (CCTA) has the potential to be a faster, simpler, substantially cheaper and more readily delivered alternative to invasive coronary angiography (ICA) and may be considered as highly effective and safe imaging strategy.

**Aim:** To assess the strategy of using coronary computed tomography angiography (CCTA) in patients with low-to-intermediate risk of ACS in relation to early (in-hospital) and long-term (18 months) prognosis in comparison with standard of care (SOC).

**Material and methods:** The study included 259 patients with low-to-intermediate non-STE-ACS (M47.9%, mean age 62.2 ± 9.4 years). Patients of group 1 (n = 148, M46.6%, 61.99 ± 9.92 yrs) underwent CCTA to assess the presence of coronary lesions, while ICA was performed in patients of group 2 (n = 111, M49.5%, 62.4 ± 8.6 yrs). Attending physician was responsible for the final decision which approach to choose. The follow-up period was 18 months.

**Results:** Both groups were comparable according to age, presence of comorbidities and main risk factors. Patients in the group 1 showed lower scores according to the GRACE (103.52 ± 14.85 vs. 109.44 ± 12.86; p < 0.001) and lower values of hs-Troponin I. However, there no patients in both groups with the dynamics of hs-Troponin I suitable for the criteria of myocardial infarction.

In the vast majority of patients from the CCTA group – 85 patients (57.4%) there was no atherosclerotic plaques within the coronary arteries; 41 patients (27.7%) had less than 50% stenosis, and in 22 patients (14.9%) – more than 50% stenosis in at least one coronary artery. 20 out of 22 patients were referred for ICA lately, of which PCI was performed in 10 patients, plus multivessel disease was detected in 2 patients, the FFR showed hemodynamically insignificant stenosis in 4 patients, and in 4 remaining patients no significant lesions were detected also.

In the group 2 patent coronary arteries were observed in 76 (68.5%) patients, hemodynamically insignificant lesions (<50%) were detected in 20 patients (12.3%), and 15 (13.5%) patients had significant lesions, of which 12 patients underwent PCI, and three patients had multivessel disease.

There was no in-hospital mortality in both groups. All-cause mortality during follow-up was 4.05% in the CCTA group, and 7.2% in the ICA group (p = 0.28). CV mortality was 0% and 0.9%, accordingly in the CCTA and ICA groups (p = 0.43) (Figure 1).

**Figure 1:**
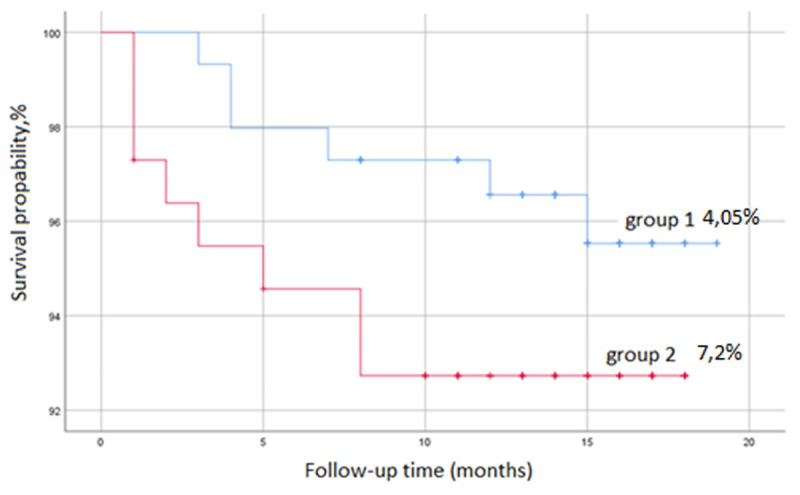
Kaplan-Meier curves for all-cause mortality in the CCTA and ICA groups.

**Conclusion:** First-line CCTA approach in patients with low- to-intermediate-risk non-STE-ACS is not inferior to the standard of care for managing these patients, while significantly reducing the need for the ICA.

Abstract ID: 485

Track: Coronary artery disease

## Pathological Q Waves in Myocardial Infarction in Patients Treated by Primary PCI in Pakistan

Khalida Soomro

Ziauddin Hospital

**Objective:** Myocardial infarction (MI) remains a leading cause of morbidity and mortality worldwide, with timely reperfusion through primary percutaneous coronary intervention (PCI) being a cornerstone in its management. This study aimed to investigate the association between pathological Q waves and infarct size in patients undergoing primary percutaneous coronary intervention (PCI) for ST-segment elevation myocardial infarction (STEMI). Additionally, the research explored whether Q-wave regression was linked to improvements in left ventricular ejection fraction (LVEF), infarct size, and left ventricular dimensions, particularly in patients with early Q-wave formation compared to those without or with persistent pathological Q waves.

**Background:** The criteria for diagnosing pathological Q waves post-acute myocardial infarction (MI) have evolved, and limited data exist regarding the correlation between Q-wave regression and preservation of LVEF in patients experiencing an initial Q-wave MI.

**Methods:** A retrospective analysis was conducted on a cohort of patients total of 1,200 STEMI who underwent primary PCI for acute MI in Pakistan between 2017 to 2022. Clinical data, electrocardiograms (ECGs), and angiographic findings were reviewed to identify the presence of pathological Q waves post-procedure. Standard 12-lead electrocardiograms (ECGs) before and after PCI, as well as at 1, 4, 12, and 24 months of follow-up. Q-wave MI was defined based on four clinical criteria: “classic” criteria, Thrombolysis In Myocardial Infarction criteria, and 2000 and 2007 consensus criteria. The primary outcome measures included the incidence of pathological Q waves, their association with procedural variables.

**Results:** The classic ECG criteria exhibited the strongest correlation with infarct size as measured by CMR. The incidence of Q-wave MI, according to the classic criteria, was 23% at 1 hour after PCI. After 24 months, 40% of patients with initial Q-wave MI displayed Q-wave regression. Patients with Q-wave MI exhibited larger infarct size and lower LVEF on baseline/ECHO CMR compared to non-Q-wave MI patients. Q-wave regression was associated with significantly larger LVEF improvement at 24 months compared to both persistent Q-wave MI and non-Q-wave MI. Additionally, the study investigates the correlation between the development of pathological Q waves and adverse cardiovascular events during follow-up, including recurrent infarctions, heart failure, and mortality.

**Conclusions:** The association of Q waves with infarct size is most pronounced when using the classic Q-wave criteria. Q-wave regression is linked to the most substantial improvement in LVEF, as assessed with CMR. This study provides valuable insights into the dynamic changes in Q waves and their implications for myocardial infarction outcomes in patients treated with primary PCI.

Abstract ID: 746

Track: Coronary artery disease

## Correlation between strain echocardiogram and coronary angiogram in patients with stable angina – a tertiary care centre based prospective cohort study

Venkatesh Raju^1^, Deepthy M S^2^

1) Thoothukudi medical college, Tuticorin, Tamil Nadu, India 2) Jawaharlal Nehru Medical College of KLE Academy of higher education and research (KAHER), Belagavi, Karnataka

**Background:** More than 50% patients referred to coronary angiogram (CAG) show normal or non -obstructive coronary artery disease (CAD) (1). Thus, we are in need of a simple, non-invasive method to improve the selection of patients who are referred for CAG. Exercise electrocardiogram has limited power to rule in and out obstructive CAD (2). Ionising radiation exposure associated with coronary CT angiogram and nuclear perfusion imaging is a concern in young individuals. Two-dimensional speckle tracking echocardiography (2D STE) is not influenced by noises or adjacent segment tethering nor is it angle dependent. Intermittent ischemia may result in subtle form of stunning that may be detectable with strain measurements. Longitudinally arranged subendocardial fibres are more vulnerable due to their direct exposure to the intraventricular blood pressure and anatomy of coronary circulation (3). Thereby, longitudinal function is first impaired in CAD, which can be detected by strain measurements. Myocardial shortening that happens after aortic valve closure is defined as post-systolic shortening (PSS) which can be measured and quantified objectively by PSI (Post-systolic index). Elevated PSI in actively contracting myocardium correlates well with viability. Therefore, the current study was designed to study the diagnostic accuracy of GLS and PSS derived from speckle tracking echocardiography to detect CAD in patients with stable angina pectoris.

**Objective:** To evaluate the diagnostic potential of resting global longitudinal peak systolic strain (GLS) and post-systolic shortening (PSS) by speckle-tracking echocardiogram to predict the presence and severity of coronary artery disease (CAD) in patients with stable angina.

**Methodology:** Single centre prospective study was conducted in sixty consecutive patients who presented with effort angina to Thoothukudi medical college. GLS, PSS and post systolic index (PSI) measurements by speckle-tracking echocardiogram was performed prior to coronary angiography (Figure 1). Significant CAD was defined by the stenosis of ≥70% stenosis in ≥1 epicardial coronary artery. The correlation between individual and combined measurements of GLS and PSI to severity of CAD was studied.

**Figure 1:**
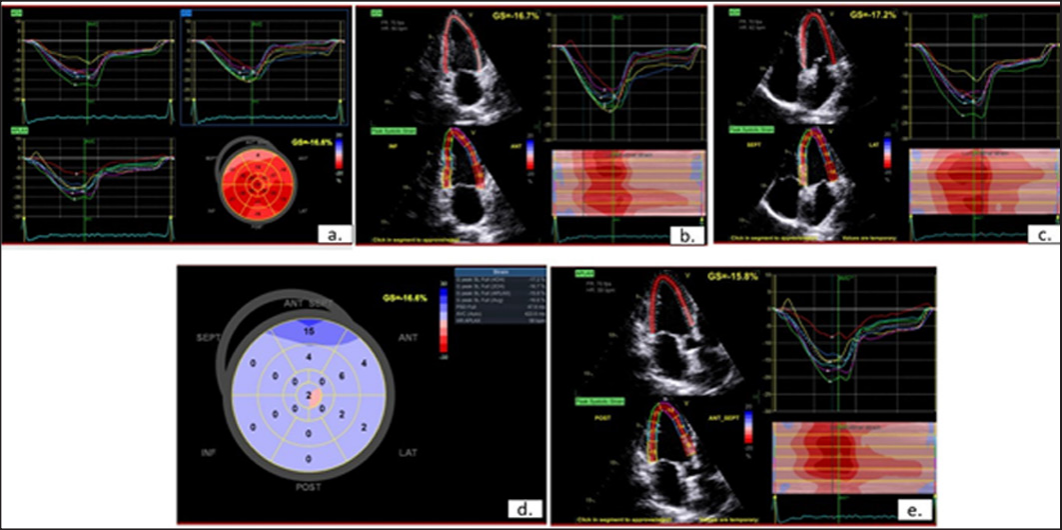
Speckle tracking echocardiogram measuring global longitudinal strain **(a, b, c, e)** and post systolic shortening **(d)**.

**Results:** There was a statistically significant difference in the median of GLS-17 segment between normal coronaries {–17.5 (–18.5, –16.4)} and significant CAD { –15.60(–17.00, –14.30)} with p value of 0.006. The GLS cut-off score of –16.85 had sensitivity of 73.7% and specificity of 61.1% to detect significant CAD with p value of 0.02 (Table 1). The regional longitudinal strain (RLS) for 17 segment showed statistically significant difference for localisation of the affected vessel for left circumflex (LCX) and right coronary artery (RCA) with p value of 0.047 and 0.008 respectively. PSS had a sensitivity of 65.8% to diagnose significant CAD.

**Table 1:** Diagnostic accuracy for GLS for predicting significant CAD.


VARIABLES	SENSITIVITY (%)	SPECIFICITY (%)	PLR	NLR	PPV (%)	NPV (%)	CUT-OFF	AUC (95% CONFIDENCE INTERVAL)	P VALUE

GLS 17 segment	73.7	61.1	1.9	0.4	80.0	52.4	–16.85	0.69(0.53, 0.85)	0.02*


GLS- Global longitudinal strain PLR-positive likelihood ratio NLR-negative likelihood ratio PPV- positive predictive value NPV- negative predictive value AUC- Area under curve.

**Conclusion:** Two-dimensional strain echocardiogram performed at rest was able to predict significant CAD instable angina patients. Thus, strain echocardiography is a non-invasive tool with good sensitivity and specificity to assess myocardial ischemia.

Abstract ID: 351

Track: CVD and women

## Exploring Sex Differences in Acute Myocardial Infarction: A 20-Year Analysis of Pakistani Patients using Traditional and Artificial Intelligence based Statistical Approaches

Zainab Samad^1,2*^, Mohummad Hassan Raza Raja^2^, Anum Ali^1^, Zarmeen Nasim^2^, Linda K. Shaw^3^, Ali Aahil Noorali^1^, Safia Awan^1^, Saadia Sattar^1^, Minaz Mawani^1^, Gerald S. Bloomfield^4,5^, Pamela S. Douglas^4^, Osman Faheem^1^, Nasir Rahman^1^, Javed Tai^1^, Zulfiqar A. Bhutta^6,7^, Salim S. Virani^8^, Adil H. Haider^9^

^1^Department of Medicine, Aga Khan University, Karachi, Pakistan, ^2^CITRIC Health Data Science Center, Aga Khan University, Karachi, Pakistan, ^3^Independent Biostatistical Consultant, ^4^Division of Cardiology, Department of Medicine, Duke University, USA, ^5^Duke Global Health Institute, Duke University, USA, ^6^Office of Research and Graduate Studies, Aga Khan University, Karachi, Pakistan, ^7^Institute of Global Health and Development, Aga Khan University, UK, Kenya, Pakistan, ^8^Center for Global Child Health, SickKids, Toronto, Canada, ^9^Dean’s Office, Aga Khan University, Karachi, Pakistan

**Background & Objectives:** There is limited data on the use of artificial intelligence, in particular readily available large language models (LLMs) to analyze large clinical datasets and undertake complex analysis, reliably and accurately. Using traditional analysis and generative artificial intelligence (AI) (Chat Generative Pre-training Transformer 4.0 (ChatGPT 4.0)), we aimed to understand sex differences in acute myocardial infarction (AMI) treatments and outcomes over twenty years at a major academic medical center in Pakistan.

**Methodology:** All patients > 18 years admitted with a primary diagnosis of AMI from January 1, 1999, to December 31, 2018, were included. Electronic clinical records were used to extract diagnosis codes. Unadjusted and adjusted logistic regression models were examined to understand the association of sex with in-hospital mortality and the association of age and AMI type. All analyses were first performed using STATA windows version 14.2 (StataCorp, College Station, TX) and then repeated using ChatGPT 4.0.

**Results:** STATA and ChatGPT 4.0 produced the same results for descriptive analysis. Among 11,673 patients with a primary discharge diagnosis of AMI, 3,683 (31.6%) were women and 5,319 (45.6%) had ST-elevation-myocardial-infarction (STEMI). Chat GPT4.0 was accurately able to exactly replicate the results of basic gender analysis, univariate and multivariate regression with minimal input. Compared with men, women were less likely to have undergone angiography (65.4% vs 52.7%; p < 0.001), percutaneous coronary intervention (PCI) (42.7% vs 32.0%; p < 0.001) and Coronary Artery Bypass Graft Surgery (10.6% vs 7.0%; p < 0.001). After adjustment for age, type of AMI, co-morbidities, PCI, and discharge year, women had a 23% higher risk of in-hospital mortality compared with men (adjusted OR: 1.23; 95% CI:1.07–1.41, p = 0.003). Interaction testing, using both STATA and ChatGPT 4.0, indicated that the risk of mortality associated with age and STEMI differed significantly for women and men. While the magnitude of the point estimates between STATA and ChatGPT 4.0 were not exactly the same (with ChatGPT 4.0 requiring multiple prompts), the differences were statistically non-significant. Interaction testing in our adjusted model, via STATA indicated the adjusted odds ratio (AOR) for an age per 10-year increase was 1.34 (95% CI:1.22–1.47) for women and 1.59 (95% CI:1.48–1.71) for men (p = 0.004); the AOR for STEMI-vs-Non-ST-elevation-MI (NSTEMI) was 3.43 (95% CI:2.76–4.27) for women and 2.46 (95% CI:2.06–2.95) for men (p = 0.016). Interaction testing in our adjusted model, via Chat GPT4.0, indicated that the AOR for age per 10-year increase was 1.33 (95% CI:1.10–1.60) for women and 1.62 (95% CI:1.51–1.74) for men (p = 0.00072); the AOR for STEMI-vs-NSTEMI was 3.56 (95% CI:2.28–5.54) for women and 2.36 (95% CI:1.98–2.82) for men (p = 0.000266).

**Conclusions:** Females, particularly those with STEMI, had worse outcomes compared with men. ChatGPT 4.0 can be used for basic statistical analyses for medium-large datasets but requires considerable critical human oversight for more complex analyses.

**Keywords:** Acute Myocardial Infarction; Sex; STEMI; NSTEMI; Disparity and Inequity; Artificial Intelligence; ChatGPT

Abstract ID: 353

Track: Digital technology and artificial intelligence

## Using Machine Learning Approaches to Predict In-Hospital Mortality in Pakistani Patients with Acute Myocardial Infarction

Zarmeen Nasim^1^, Mohummad Hassan Raza Raja^1^, Zainab Siddiq^2^, Zainab Samad^1,2*^

^1^ CITRIC Health Data Science Center, Aga Khan University, Karachi 74800, Pakistan. ^2^ Department of Medicine, Aga Khan University, Karachi 74800, Pakistan.

**Background & Objectives:** Traditional, in-hospital mortality risk prediction scores for acute myocardial infarction (AMI) patients are based on multivariate logistic regression approaches. They are limited in the number of variables they contain and their inability to account for non-linear and complex relationships between variables. Machine Learning (ML) algorithms have the potential to overcome these limitations, however, as yet have been tested on datasets originating from high income countries. This study aims to build and evaluate the utility of ML models to predict in-hospital mortality among patients presenting with AMI, at a tertiary care hospital in Pakistan.

**Methodology:** 8579 patients with a primary discharge diagnosis of AMI between January 2007 and December 2018 admitted at a tertiary care hospital in Karachi were included in the study. Patient variables included demographics, comorbidities, length of stay, vitals, laboratory reports, inpatient procedures, drugs, past medical procedures, and in-hospital mortality. Two types of models were trained using ML algorithms, with the algorithms differing by the number of attributes used in the training phase. For the larger model, a subset of the dataset comprising 23 attributes was selected to train the model, whereas the simpler model was trained using a set of 11 attributes immediately available when the patient arrives at the hospital. For both models, the dataset was randomly distributed into training set (70%) and test set (30%) and a correlation analysis was performed to identify removal of highly correlated attributes. Two features (Body Mass Index (BMI) and BMI category) were engineered using height and weight variables. After feature engineering, four ML algorithms (three advanced: Random Forest, Gradient Boosting, XGBoost and one basic: Logistic Regression (representing traditional risk prediction scores)) were used to train the models. The performance of the four algorithms was evaluated on the test dataset using specificity, sensitivity, f-measure, area under the curve (AUC) for receiver operating characteristic (ROC) and precision-recall (PR) curves. Differences in performance between the algorithms was tested via the Kruskal–Wallis test. For the Gradient Boosting algorithms, a permutation-based method was used for computing variable importance using feature importance scores.

**Results:** Figure 1 displays ROC and PR curve for the larger and simpler models. For the larger model, Logistic Regression and Gradient Boosting algorithms achieved the maximum AUC-ROC score (AUC-ROC = 0.89, 95% Confidence Interval (CI) (0.87, 0.91)) and F1 score (0.91). The differences between the four algorithms were non-significant (p > 0.05). For the simpler model, the Gradient Boosting algorithm outperformed the other machine learning algorithms (AUC-ROC = 0.784, 95% CI (0.76, 0.81), F1 Score = 0.87), however the simpler model algorithm differences were non-significant (p > 0.05). The AUC-ROC differences between the best performing algorithms (Gradient Boosting) between the larger and simpler models were also non-significant (p > 0.05). In the larger model using the Gradient Boosting Algorithm the three most importance variables were as follows: cardiogenic shock (Score = 0.066), patient on ventilator (Score = 0.029), acute kidney failure (Score = 0.023). In the simpler model, creatinine (Score = 0.140), type of AMI (Score = 0.071), age (Score = 0.027) were the most important predictors.

**Figure 1:**
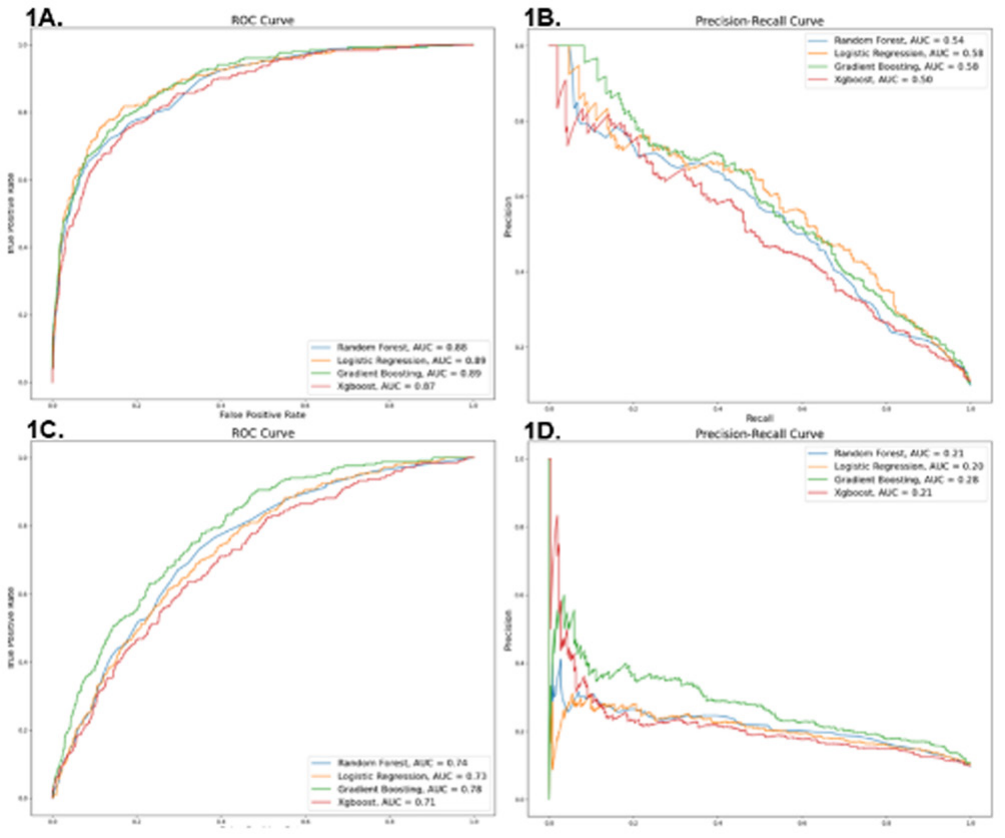
**A.** ROC curve: Larger Model Algorithms, **B.** PR curve: Larger Model Algorithms, **C.** ROC curve: Simpler Model Algorithms, **D.** PR curve: Simpler Model Algorithms.

**Conclusion:** Our results show that on a robust complete dataset, with limited variables, advanced ML algorithms do not outperform traditional logistic regression based approaches in predicting in-hospital mortality for AMI patients. This has significant implications, especially for lower resource settings, with limited data infrastructure and ML expertise. However, it can be hypothesized that with incomplete datasets, common in lower resource settings due to human, technical and workflow constraints, ML algorithms are likely more adept in imputing data, overcoming missing data values, providing more accurate predictions.

Abstract ID: 1163

Track: Environmental health

## A cluster randomised controlled trial to evaluate effectiveness of a self-help group intervention to encourage smoke-free homes in slums of Kochi, Kerala, India

Aswathy S*, Vijayakumar K, Sreelakshmi Mohandas, Navami S, Jaideep Menon

*Amrita Institute of Medical Sciences

**Background and Objectives:** Globally, tobacco smoking including second-hand smoke (SHS), is one of the three leading risk factors for global disease burden, others being high blood pressure, and household air pollution from solid fuels (1). Though, there is a decline in the proportion of people exposed to domestic SHS in India, according to the recent Global Adult Tobacco Survey-2 (GATS-2) report, it continues to be high at 38.2% among adults (2). Being female, illiterate, poor, homemaker, resident of the rural areas was associated with the highest exposure to SHS at home in GATS-2. The objectives of the study were To determine the effectiveness of an intervention led by women’s self-help groups to reduce indoor tobacco smoking by measuring urinary cotinine levels and reduction in indoor smoking by measuring PM 2.5 as an indicator of tobacco smoke in the home environment.

**Methods:** A cluster-randomized community-based trial was carried out. Clusters were slums with a population of more than 400 or 100 households in the Kochi Corporation and adjacent peri-urban areas. 30 clusters were selected randomly from the sampling frame of 74 slums and colonies with a population of more than 400 population from a total list of 300 slums and colonies of Kochi Municipal Corporation and adjacent areas. They were randomly assigned by a computer-generated table to the intervention or control arm on a 1:1 allocation ratio. All individuals who were living in the area for more than 6 months were eligible to participate in the study. In the households the individuals were screened to identify homes where the male members are between 18–80 years and smoke within the homes. Informed written consent was obtained from willing participants. Four semi-structured questionnaires were pretested and administered in the household and for smoker, spouse and child. Blood pressure of the smoker and spouse was measured by an electronic sphygmomanometer in a seated position after ensuring that the person is relaxed. If the reading is ≥140/90 mmHg the measurement will be repeated after 30 min and the average of the two will be taken. The urine cotinine level was measured with the help of Cal biotech’s ELISA kit which measured cotinine levels by ELISA (solid phase competitive ELISA). SHS was measured through the air pollution monitor within the home with ATMOS devise which measure particulate matter of size 2.5 microns and is a marker for the presence of SHS in the home. The device was charged and left in the home for recording for 6–8 hours at an elevated place. The machine recorded PM 2.5, 10 PM. The baseline was followed by the first post-intervention 8 months later and then the next 6 months later. Thus, 2143 families were surveyed at baseline in intervention and 2453 in control with 8318 members and 8757 members to obtain 331,326 willing smoker participants household in intervention and control respectively. Some preliminary results are presented here.

**Results:** The mean age of the study participants (smokers) in both the groups were 53.2 (15.22) and 52.6 (11.53) yrs respectively. The follow up values of urine cotinine at 8 months and the next 6 months later. There was no decline of cotinine levels at first post intervention, though by the second post intervention there was a decline in cotinine, though the reduction was not significant. Particulate matter 2.5 showed a significant decline from the first post intervention onwards and it persisted till the second intervention. The same was observed for PM 10 as well (Table 1).

**Table 1:** Cotinine levels, PM 2.5 and PM 10 levels following intervention.


	MEDIAN DIFF FROM BASELINE TO POST 1	P	DIFF FROM BASELINE TO POST 2	P

Cotinine among spouse **Intervention(158)**	66.79(–12.4, 100)		75(–**19.96, 100)**	

Control(120)	66.00(–30.69, 98.68)	0.31	66.66(–10.52, 98.18)	0.54

Cotinine among children Intervention(35)	41.6(–184.9, 92.43)	0.23	100(16.6, 100)	0.32

Control(13)	63.49(20.34, 94.66)		81(65, 100)	

**PM2.5** lntervention(n = 311)	47.21(–14.7, 70)	.000	68.5(32.08, 85.89)	.000

Control(n = 319)	–21.14(–87.15, –17.7)		–41.62(–211.42, –41.62)	

**PM 10** lntervention(n = 310)	42.56(–20, 66.4)	.000	67.79(20.16, 85.05)	.000

Control(n = 297)	–10.4(–79.87, 19.93)		–13.7(162.94, 49.98)	


**Conclusion:** Thus, a self-help group intervention can help to reduce the particulate 2.5 pm. However, more studies are necessary with bigger sample size to determine the impact on cotinine.

Abstract ID: 481

Track: Environmental health

## Impact of China Pakistan Economic Corridor on physical activity, obesity and hypertension in Himalayan Mountain villages of Pakistan

Syed M. Shah

College of Medicine and Health Sciences, United Arab Emirates University, Al Ain, UAE

**Background/Introduction:** China Pakistan Economic Corridor (CPEC) is part of the One Belt One Road initiative engaging 64 countries along the course of the ancient Silk Road, has improved access to modern mood of transportation to villagers in Himalayan mountain villages of Pakistan. Few data are available on the impact of CPEC on physical inactivity and hypertension.

**Purpose:** This study evaluated the prevalence of hypertension in CPEC villages and non-CPEC villages in Gilgit, Pakistan.

**Methods:** We selected 6 villages on CPEC road and 6 villages away from CPEC road. We examined the prevalence of hypertension (home-based blood pressure measurement ≥140/90 mmHg) in a random sample of adults aged ≥18 year in CPEC villages (n = 610) and Non-CPEC villages (n = 572) using automated blood pressure monitors. Trained nurses measured weight and height using SECA (Germany) weight and height scales. A body-mass index (BMI) of ≥25 and 30 kg/m^2^ used to define overweight and obesity. Less than 30 minutes of moderate physical activity for 5 days per week or 20 minutes of vigorous activity for 3 days per week on International Physical Activity Scale considered insufficiently active.

**Results:** In total, 1182 participants were eligible for analysis (56.3% male, mean age: 44.8 ± 14.5 in CPEC villages and 52.9% male, mean age: 41.6% ± 15.1 in non-CPEC villages. A high proportion (29.5%) were insufficiently active in CPEC-villages as compared to non-CPEC villages (16.9%). In CPEC villages 32.5% overweight, 11.7% obese and 34.3% had hypertension, compared to non-CPEC villages with 21.6% overweight, 6.8% obese and 23.9% had hypertension. After controlling for age, gender and education in a multivariable logistic regression analysis, study participants from CPEC villages were more likely to have hypertension (adjusted odds ratio (AOR) = 1.57; 95% CI 1.07–2.32), higher BMI (AOR = 1.05; 95%CI 1.03–1.27) and insufficiently active (AOR = 1.86 (1.27–2.74).

**Conclusion:** People living in CPEC villages are more vulnerable to be physical inactivity, obese and to have a higher prevalence of hypertension.

Abstract ID: 102

Track: Fixed-dose combination therapies and polypill

## Global expert consensus endorsing the cardiovascular polypill strategy with acetyl salicylic acid, atorvastatin and ramipril as baseline treatment post-cardiovascular events based on strong recent evidence

Daniel Piñeiro^1^, José Ramón González Juanatey^2^, José María Castellano Vázquez^3^, Carlos^7^, Ignacio Ponte-Negretti^4^, Ana Abreu^5^, Francisco Araújo^6^, Alexander Parkhomenko^7^, Enrique^8^, Gómez Alvarez^8^, Burkhard Weisser^9^, Álvaro Sosa-Liprandi^10^, Valentín Fuster^11^

1) Hospital de Clínicas “José de San Martín”, Buenos Aires, Argentina; Faculty of Medicine, University of Buenos Aires, Argentina; World Heart Federation, Geneva, Switzerland, 2) Hospital Clínico Universitario; Instituto de Investigación Sanitaria (IDIS), Santiago deCompostela, Galicia; Centro de Investigación Biomédica en Red Enfermedades Cardiovasculares (CIBERCV), Madrid; Spain, 3) Centro Nacional de Investigaciones Cardiovasculares Carlos III (CNIC), Madrid, Spain; HM, Hospitals, Madrid, Spain, 4) Instituto Médico La Floresta, Caracas, Venezuela, 5) Centro de Reabilitação Cardiovascular Hospital de Santa Maria, Centro Hospitalar Universitário, Lisboa Norte, Centro Académico de Medicina de Lisboa; Instituto de Medicina Preventiva e, Instituto de Saúde Ambiental da Faculdade de Medicina da Universidade de Lisboa, Lisbon, Portugal, 6) Hospital Lusíadas, Lisbon, Portugal, 7) National Scientific Centre named after Strazhensku (former Insitute of Cardiology), Kyiv, Ukraine, 8) Centro Médico 20 de Noviembre, Mexico City, Mexico, 9) Institute of Sports Science Christian Albrechts; University of Kiel, Kiel, Germany, 10) Sanatorio Güemes, Buenos Aires, Argentina; Inter-American Society of Cardiology, Mexico City, Mexico, 11) Fundación Centro Nacional de Investigaciones Cardiovasculares Carlos III (CNIC), Madrid, Spain; Icahn School of Medicine at Mount Sinai, New York, US

**Background & Objectives:** In 2019, cardiovascular disease (CVD) caused 10.8 million deaths in Asia (about 35% of all deaths) (1). After initial myocardial infarctions (MI), 14.3% of Asian patients experienced recurrent major adverse cardiovascular events (MACE) within the first year, including 5.7% cardiovascular (CV)-related deaths. The multinational, randomized, controlled clinical trial SECURE showed that the CV-polypill strategy (acetyl salicylic acid [ASA]+atorvastatin+ramipril) reduces CV mortality by 33% in acute MI patients compared to standard care over a 3-year (median) follow-up (2). Based on this evidence, the CV-polypill has been included in the World Health Organization essential medicines list for 2023 to effectively and affordably address a critical global healthcare need (3). The 2023 ACS ESC guidelines recommend using a polypill to enhance CVD outcomes and adherence (4). The aim of this global consensus among medical experts was to agree on crucial implementation aspects for the CV-polypill strategy (ASA+atorvastatin+ramipril) as a baseline treatment for preventing recurrent CV events (CVe) in routine clinical practice.

**Methods:** A two-round modified Delphi method which involved creating and validating a questionnaire consisting of 30 evidence-based statements was applied. This process was guided by input from 8 renowned cardiologists. The Delphi panel, comprising 50 physicians from 19 countries across Asia, Latin America, and Europe, used a three-point Likert scale to assess their agreement and perceived importance regarding the statements. Consensus was achieved when ≥80% agreement or recognition as ‘very important’ or ‘important’ was reached. Statements lacking consensus in the first round were refined in the second round based on evidence and feedback from participants. Persistent disagreements were resolved through a face-to-face meeting with experts. Descriptive statistics were utilized for analysis.

**Results:** Response rates for rounds 1 and 2 were 76% and 74%, respectively. 82% of panellists were cardiologists and 13%, internists; 74%, frequently prescribed the CV-polypill strategy. Consensus was achieved on 93.3% of statements. 97.4% of panellists agreed that employing the CV-polypill strategy could reproduce a 24% relative risk reduction in recurrent MACE over 3 years (median) after a MI compared to administering cardioprotective drugs separately at equal safety (97.4%). Unanimous consensus (100%) was reached on initiating the CV-polypill strategy as baseline therapy upon hospital discharge or at the first follow-up visit. 81.1% supported early initiation. Robust agreement (97.3%) existed on employing algorithms for initiating and transitioning to the CV-polypill strategy from separate cardioprotective drugs, considering patient preferences (97.5%). The primary treatment objectives at hospital discharge agreed were achieving therapeutic goals (81.6%) and enhancing adherence (94.7%). 89.5% of panellists convened that the CV-polypill strategy delivers cost savings over a patient’s lifetime by reducing CVe, hospitalizations, and productivity losses. Panellists agreed on the efficacy and safety of the CV-polypill strategy on stroke (94.7%), peripheral artery disease (92.1%), type 2 diabetes mellitus (81.6%), and both genders (84.2%). Unanimous agreement (100%) confirmed an increase in patient satisfaction with the CV-polypill simplified regimen; 94.7% emphasized its convenience. 97.4% recognized the potential for 10–17% higher patient adherence with the CV-polypill compared to separate cardioprotective drugs in routine practice. Number of tablets taken per day (80%) and staff explanation of the CV-polypill strategy (71.4%) were the most important factors affecting patient adherence on hospital discharge. Effective doctor–patient communication (100%) and patient’s willingness to persist on medication (94.6%) were deemed critical factors for the successful implementation of the CV-polypill strategy at home.

**Conclusions:** Experienced medical specialists from various continents reached a consensus on vital implementation aspects and strongly advocate the prompt adoption of the CV-polypill strategy (ASA+atorvastatin+ramipril) in routine clinical practice to reduce CVD recurrence, improve treatment outcomes and prognosis, and potentially lower CVD-related costs following a CVe.

Abstract ID: 1113

Track: Heart failure

## The epidemiology and risk factors for myocarditis: an Australian population-linkage cohort study

Timothy Kwan, Gemma Kwan, David Brieger, Leonard Kritharides, Vincent Chow, Austin Chin Chwan Ng

The University of Sydney

**Background & Objective:** Myocarditis is defined as myocardial inflammation and is an important contribution to the global burden of cardiovascular disease with both acute and chronic sequelae (1). Myocarditis has received increased attention following the Covid-19 pandemic with both Covid-19 infection and vaccination recognized as risk factors for myocarditis (2). However, myocarditis is a diverse disease with multiple possible etiologies.

We aimed to better characterize the diverse cohort of patients with myocarditis and quantify risk factors for disease and recurrence.

**Methodologies:** In this cohort study, data were linked from the New South Wales (NSW) Admitted Patient Data Collection (APDC) and the NSW Registry of Births, Deaths & Marriages. Admissions dating between 2001 and 2022 were included. Patients were included in the present study if they had a diagnosis of myocarditis recorded according to the International Statistical Classification of Diseases and Related Health Problems, Tenth Revision, Australian Modification (ICD-10 AM) codes, either as a primary or secondary issue during a hospital admission.

The incidence of myocarditis adjusted for age and sex was quantified. The classification of myocarditis was classified as either autoimmune, malignant, ischemic, or reactive. Autoimmune, malignant, and ischemic myocarditis were defined as having these conditions as a primary or secondary diagnosis during admission. Reactive myocarditis was defined as having an admission for a respiratory or digestive system condition in the 30-days prior to the myocarditis admission.

Risk factors for myocarditis were quantified by conditional logistic regression by comparing the month prior to the diagnosis of myocarditis with the preceding 12 months.

**Results:** There were 4,071 patients diagnosed with their first episode of myocarditis or 5,968 unique admissions. The incidence of myocarditis in NSW rose from 2.6 per 100,000 in 2001 to 8.6 per 100,000 persons in 2022 (Figure 1).

**Figure 1:**
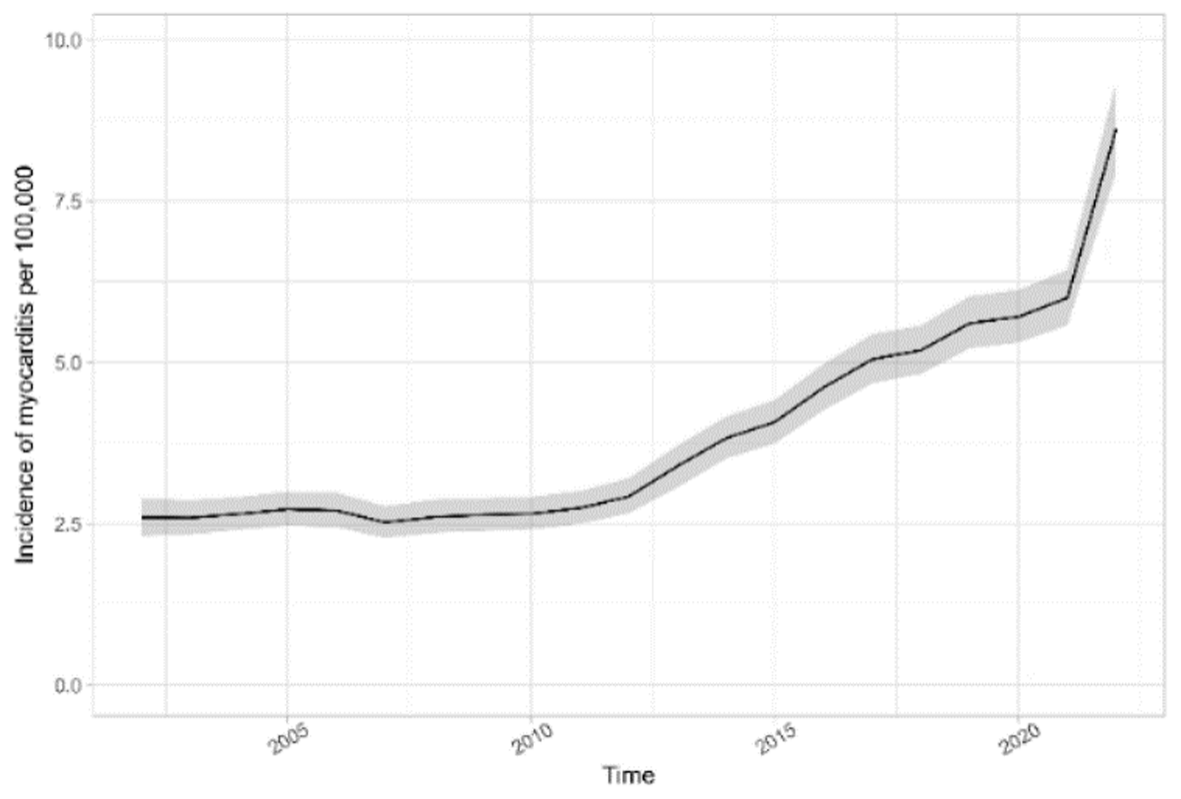
Age standardized incidence of myocarditis admissions over time.

A large proportion (38%) of patients had a reactive class of their first diagnosis of myocarditis. Despite Covid-19 being only a recently diagnosed disease, 6.9% of patients with myocarditis presented within 30 days of a diagnosis of Covid-19. This exceeded the proportion with other upper respiratory tract infections such as influenza (2.5%). Malignancy was found in 6% of patients with myocarditis. Autoimmune diseases including sarcoidosis and systemic lupus erythematosus were found in 6% of patients with myocarditis. Although myocardial infarction is typically considered as exclusionary for a diagnosis of myocarditis, 16% of patients with myocarditis had a diagnosis of a myocardial infarction (Figure 2). Fifty-one percent of myocarditis could be classified as autoimmune, malignant, ischemic or reactive.

**Figure 2:**
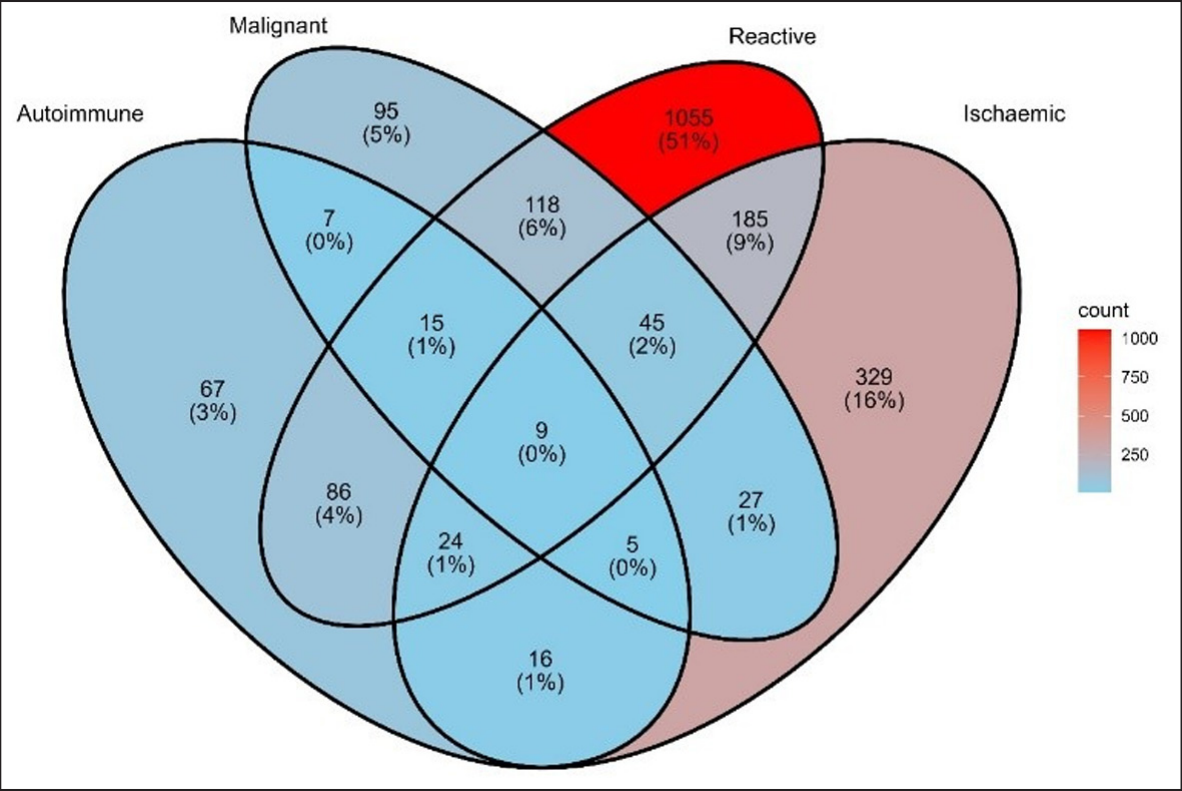
Frequency of myocarditis according to possible classifications (patient with identifiable class n = 2,083).

Diagnoses of myocarditis were much more likely to be preceded in the previous month by a hospital presentation for myocardial infarction (odds ratio [OR] 38), sarcoidosis (OR 23), pericarditis (OR 21), heart failure (OR 13), Covid-19 (OR 10), respiratory disease (OR 8.4), atrial fibrillation (OR 8.3), digestive disease (OR 3.6) or malignancy (OR 2.4) (all p < 0.0001).

**Conclusion:** Myocarditis has a diverse range of presentations but is strongly associated with respiratory illness, especially Covid-19, autoimmune disease, and malignancy. Despite myocarditis being often considered mutually exclusive with myocardial infarction, myocarditis was also strongly associated with ischemic heart disease.

Abstract ID: 103

Track: Heart failure

## The use of exogenous phosphocreatine in patients with chronic heart failure in the Russian observational study By heart

Alfiya Safiullina, Sergey Tereshchenko

Federal State Budgetary Institution National Medical Research Center of Cardiology named after academician E.I.Chazov of the Ministry of Health of the Russian Federation

**Background and objective:** CHF is based on a violation of energy metabolism, regardless of etiology, namely a deficiency of energy substrates – adenosine triphosphate (ATP), phosphocreatine and creatine kinase, which leads to a malfunction of the myocardium. Optimal drug therapy of CHF blocks neurohormonal activation, but does not affect the energy metabolism of cardiomyocytes. In this connection, the use of adjuvant course intravenous administration of exogenous phosphocreatine in addition to OMT in patients with CHF is relevant. The aim of the study was to study the efficacy and safety of intravenous infusion of exogenous phosphocreatin (EF) in patients with chronic heart failure (CHF).

**Methodologies used:** The all-Russian prospective observational study BYHEART included 842 patients who underwent an intravenous course of EF treatment. Before and after the course of EF therapy, the following studies were conducted: a questionnaire on the Minnesota Quality of Life Questionnaire for patients with CHF (MHFLQ), transthoracic echocardiography with an assessment of the left ventricular ejection fraction (LVEF), a 6-minute walk test, determination of NT-proBNP, glomerular filtration rate (GFR) and the needs of patients with coronary heart disease in nitroglycerin. All patients before the course of intravenous infusion of EF received long-term optimal drug therapy (OMT) of CHF according to national clinical guidelines.

**The results of the study:** Of the 842 patients included in the study, 60.6% (n = 510) were male. The average age was 62.4 ± 12.2 years. Of the entire cohort of CHF patients, 388 patients had FC II (46.1%), 418 – FC III (49.6%), FC IV – 36 (4.3%). Against the background of the course of EF infusion, a significant improvement in the quality of life was noted (from 61.26 ± 18.54 points to 32.61 ± 18.22 points, p < 0.05), an increase in the distance of the 6-minute walk test (from 276 ± 88 m to 333 ± 90 m, p < 0.05), LVEF (from 42.16 ± 6.32% up to 44.15 ± 6.18%, <0.05), decrease in the concentration of NT-proBNP (780 [368; 1387] pg/ml to 606 [268; 1155] pg/ml, p < 0.05), as well as a decrease in angina attacks (from 2.08 ± 1.14/day to 1.18 ± 0.95/day, p < 0.05) and the need for nitroglycerin (from 2.19 ± 1.59 tablets/day to 1.3 ± 1.18 tablets/day, p < 0.05) in patients with coronary heart disease In relation to the safety profile of EF, there was no deterioration in renal function (GFR from 65 ± 19 to 67 ± 18 ml/min/1.73 m2, p < 0.05).

**Conclusions of the study:** The results obtained demonstrate that intravenous infusion therapy with phosphocreatin is associated with an improvement in quality of life, an increase in the test distance of 6 minutes walking, an increase in LV EF and a decrease in the concentration of NT-proBNP and is not associated with a deterioration in renal function, and is also associated with a decrease in the number of angina attacks and a decrease in the need for nitroglycerin in patients with coronary heart disease.

Abstract ID: 37

Track: Heart failure

## Coronary microvascular dysfunction and heart failure with preserved ejection fraction: from bench to bedside

Kristina Kopeva^1^, Elena Grakova^1^, Alina Maltseva^1^, Andrew Mochula^1^, Anna Gusakova^1^, Andrew Smorgon^1^, Anastasiia Van-Tin-Gao^2^, Konstantin Zavadovsky^1^

^1^Cardiology Research Institute, Tomsk National Research Medical Center, Russian Academy of Sciences, Tomsk, Russia, ^2^Eastern Institute of Technology, Napier 4112, New Zealand

**Objective:** To provide with the comprehensive characteristics of coronary microvascular dysfunction (CMD) in patients with non-obstructive coronary artery disease (CAD) and to assess its prognostic value in heart failure with preserved ejection fraction (HFpEF) during 12 month follow-up period.

**Methods:** A total of 112 patients with non-obstructive CAD and preserved LVEF were enrolled. Serum levels of biomarkers were analyzed by ELISA. Two-dimensional transthoracic echocardiography with evaluation of LV diastolic dysfunction (DD) and global longitudinal strain (GLS) were performed baseline. CMD was defined as the myocardial flow reserve (MFR) ≤2 evaluated by dynamic CZT-SPECT.

**Results:** Patients were divided into groups depending on the presence of CMD: group 1 (n = 42) comprised patients with CMD, and group 2 (n = 70) included patients without CMD. Patients in group 1 were more likely to be diagnosed with HFpEF (p < 0.001), had a history of T2DM (p = 0.007) and were more often smokers (p = 0.009) than patients in group 2. The lateral e′ values were lower by 35% in group 1 (p = 0.009) than in group 2. The peak rate of tricuspid regurgitation was higher by 12% (p = 0.011), the E/e′ ratio was higher by 21.4% (p = 0.041) and LAVI by 51.2% (p = 0.038) in group 1 than in group 2. In group 1, the absolute value of GLS was lower by 29.7% (p = 0.005) than in group 2 (–14.7 [–12.9; –16.9] and –20.9 [16.1; 21.6]%, respectively). In group 1, MFR values were lower by 48.3% (p < 0.001) than in group 2 (1.39 [1.11; 1.96] and 2.69 [2.15; 3.78], respectively). In group 1, rest-MBF was higher by 30.1% (p < 0.001) and stress-MBF was lower by 30.1% (p < 0.001) compared to group 2. In group 1 the levels of soluble ST2 were 33.67 (27.65; 38.9) ng/mL, when in group 1 they were 27.5 (21.78; 30.09) ng/mL (p < 0.001). The levels of NT-proBNP were 2.6-hold greater (p = 0.004) in group 1 than in group 2 (404.2 [249.5; 1533.4] vs. 156.3 [135.26; 274.7] pg/mL, respectively). TIMP-1 levels were 2.3-hold higher (p = 0.011) (287.4 [107.38; 371.8] and 123.64 [58.66; 232.9] ng/mL, respectively) and MMP-9 levels were 1.9-hold greater (p = 0.012) (2109 [1145.7; 3235] and 1104 [721.5; 1731.9] ng/mL) in group 1 compared to group 2. Despite the values of high sensitivity C-reactive protein (hsCRP) and interleukins did not exceed the reference intervals, the differences were found in their levels. Thus, hsCRP concentrations were higher by 1.8 times (p = 0.011) in 1 group compared to group 1 (4.1 [3.0; 11.4] and 2.3 [1.1; 8.7] g/L). Interleukin-6 levels did not differ significantly between groups (p = 0.842), while interleukin-10 concentrations were lower by 21.7% (p = 0.048) in group 1 than in group 2, and interleukin-1β was 2.7-fold higher (p = 0.046) in group 1 compared to group 2. In group 1 the levels of vascular endothelial growth factor were 487.4 (107.38; 771.8) pg/mL, when in group 2 they were 223.64 (158.66; 332.9) pg/mL (p = 0.012). The endothelin-1 concentrations were 8.98 (5.65; 11.9) ng/mL in group 1, and 4.75 (2.78; 5.09) ng/mL I group 2. The levels of cardioprotective hormone catestatin were 1.8-hold lower in group 1 compared to group 2 (134.9 [112.67; 164.8] µg/mL vs. 236.98 [200.2; 310.37] µg/mL, respectively). During the 12 months of follow-up, 25 patients had the adverse outcomes: 4 patients were firstly diagnosed with HFpEF and 21 patients developed HFpEF progression (Figure 1). The incidence of adverse events preponderated in patients with CMD than in patients without it. The levels of MFR ≤1.62 and NT-proBNP ≥760.5 pg/mL were identified as cut-off values to predict adverse outcomes. Kaplan–Meier analysis (Fig. 2) showed that a rate of the adverse outcomes was significantly (p < 0.001) higher in patients with CMD (45.2%, n = 19) than in patients without it (8.6%, n = 6). Conclusion: CMD was associated with severe DD, subtle LV systolic function impairment, and overexpression of the biomarkers of fibrosis, endothelial dysfunction and inflammation, and hypoexpression of cardioprotective hormone catestatin. The incidence of HFpEF development and progression preponderated in patients with CMD than in patients without it.

Abstract ID: 38

Track: Heart failure

## Copeptin and catestatin as novel biomarkers of heart failure with preserved ejection fraction

Kristina Kopeva^1*^, Elena Grakova^1^, Anna Gusakova^1^, and Konstantin Zavadovsky^1^ Anastasiia Van-Tin-Gao^2^

^1^Cardiology Research Institute, branch of the Federal State Budgetary Scientific Institution “Tomsk National Research Medical Center of the Russian Academy of Sciences”, Tomsk, Russia; ^2^Eastern Institute of Technology, Napier, New Zealand

**Objective:** The objective of the study was to evaluate the diagnostic values of catestatin and copeptin in heart failure with preserved ejection fraction (HFpEF) and their relation to the parameters of heart rate variability (HRV) and diastolic dysfunction in patients with non-obstructive coronary artery disease (CAD).

**Methods:** A total of 83 patients (age of 62.0 [57.0; 68.5] years) were prospectively enrolled in the study. Echocardiography was performed according to a standard protocol. HRV was assessed by 24-hour ECG monitoring. Serum levels of biomarkers were evaluated by enzyme-linked immunoassay. Myocardial flow reserve estimates were obtained by CZT-SPECT.

**Results:** Patients were divided into groups depending on the presence of heart failure: HFpEF+ group (n = 63), and HFpEF- group (n = 20). High-sensitivity C-reactive protein (hsCRP) concentrations were 1.9-fold higher (p = 0.023) in HFpEF+ group compared to HFpEF- group 2. Interleukin-10 concentrations were 1.7-hold lower (p = 0.010) in patients with HFpEF than in those without it, and interleu-kin-1β was 2.7-hold higher (p = 0.046) in HFpEF+ group compared to HFpEF- group. The levels of NT-proBNP were 8.4-hold higher (p < 0.001), the serum levels of soluble ST2 were higher by 31.6% (p < 0.001) in HFpEF+ group compared to group 2. The serum concentrations of copeptin were lower by 26.2% (p = 0.023), when catestatin levels were lower by 43.1% (p < 0.001) in patients with HFpEF than in those without it. Based on ROC-analysis, the values of copeptin ≤0.334 ng/mL (AUC = 0.675; sensitivity of 66.67%; specificity of 94.74; p = 0.022) and catestatin ≤132.83 µg/mL (AUC = 0.884; sensitivity of 70.59%; specificity of 93.33; p < 0.001) were determined as cut-off values associated with the presence of HFpEF (Figure 1). The concentrations of catestatin opposite depended on NYHA functional classes (p < 0.001): patients with NYHA I functional class had the highest levels of catestatin – 189 (292; 104) µg/mL, when patients with III functional class had the lowest one – 58 (43; 132) µg/mL. The levels of catestatin in patients with NYHA II functional class were 141 (87; 209) µg/mL. The concentrations of copeptin were found (p = 0.191) to be negligible (Figure 2). Catestatin and copeptin had positive correlative link between one another (r = 0.424; p = 0.023), but negative one with NT-proBNP, soluble ST2, interleukin-1β, and hsCRP levels. Moreover, the se-rum values of copeptin were more linked with heart rhythm variability parameters, when catestatin correlated with majority of LV remodeling estimates. The positive correlation were found between copeptin and stress-myocardial blood flow (r = 0.345; p = 0.017), as well as between catestatin and myocardial flow reserve (p = 0.301; p = 0.012). Multivariate regression analysis showed that the presence of diastolic dysfunction (OR 3.31; 95% CI 2.26–5.64; p < 0.001), the overexpression of NT-proBNP ≥ 263.35 pg/mL (OR 2.56; 95% CI 2.26–8.98; p = 0.021), soluble ST2 ≥ 31.4 ng/mL (OR 1.37; 95% 1.45–12.65; p = 0.025), decreased MFR ≤2.27 (OR 1.78; 95% CI 1.32–6.43; p = 0.001), decreased levels of catestatin ≤132.83 µg/mL (OR 2.95; 95% CI 1.76–8.09; p < 0.001) were independent factors associated with HFpEF in patients with non-obstructive CAD. During 12 months of follow-up 11 patients had adverse outcomes. Based on ROC-analysis, the values of catestatin ≤136.7µg/mL (AUC = 0.964; p < 0.001) were determined as cut-off values predicted the adverse outcomes in patients with non-obstructive CAD during 12-month follow-up period (Figure 3).

**Figure 1:**
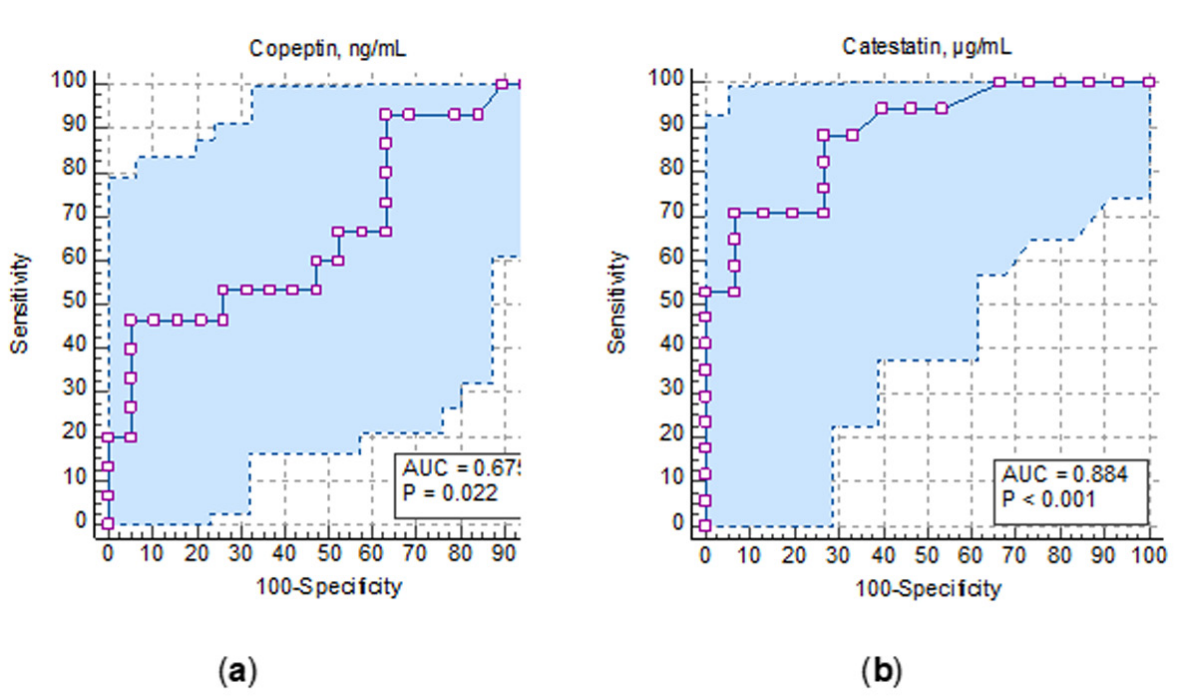
The ROC-curves of copeptin and catestatin levels associated with HFpEF: **(a)** the levels of copeptin associated with HFpEF; **(b)** the levels of catestatin associated with HFpEF.

**Figure 2:**
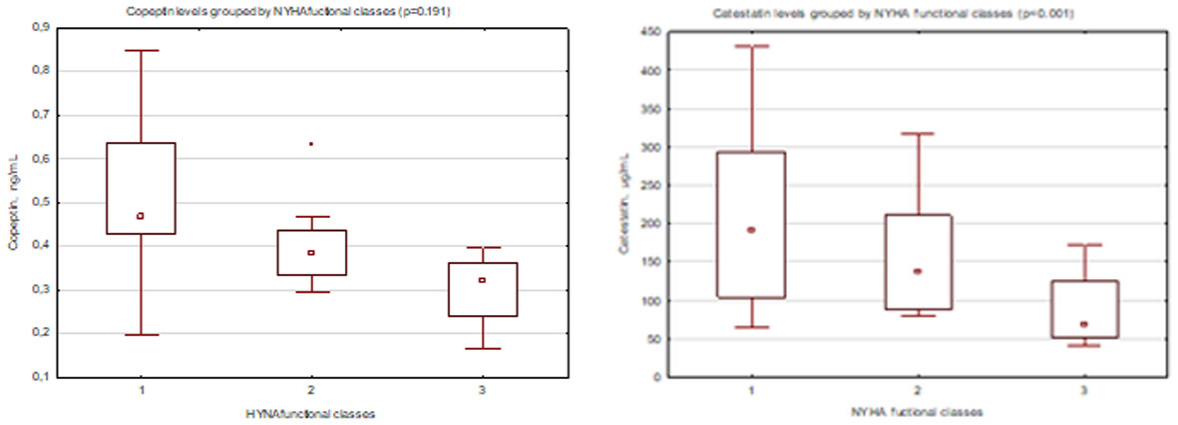
The levels of biomarkers depending on NYHA functional classes: **(a)** copeptin (p = 0.191); **(b)** catestatin (p < 0.001).

**Figure 3:**
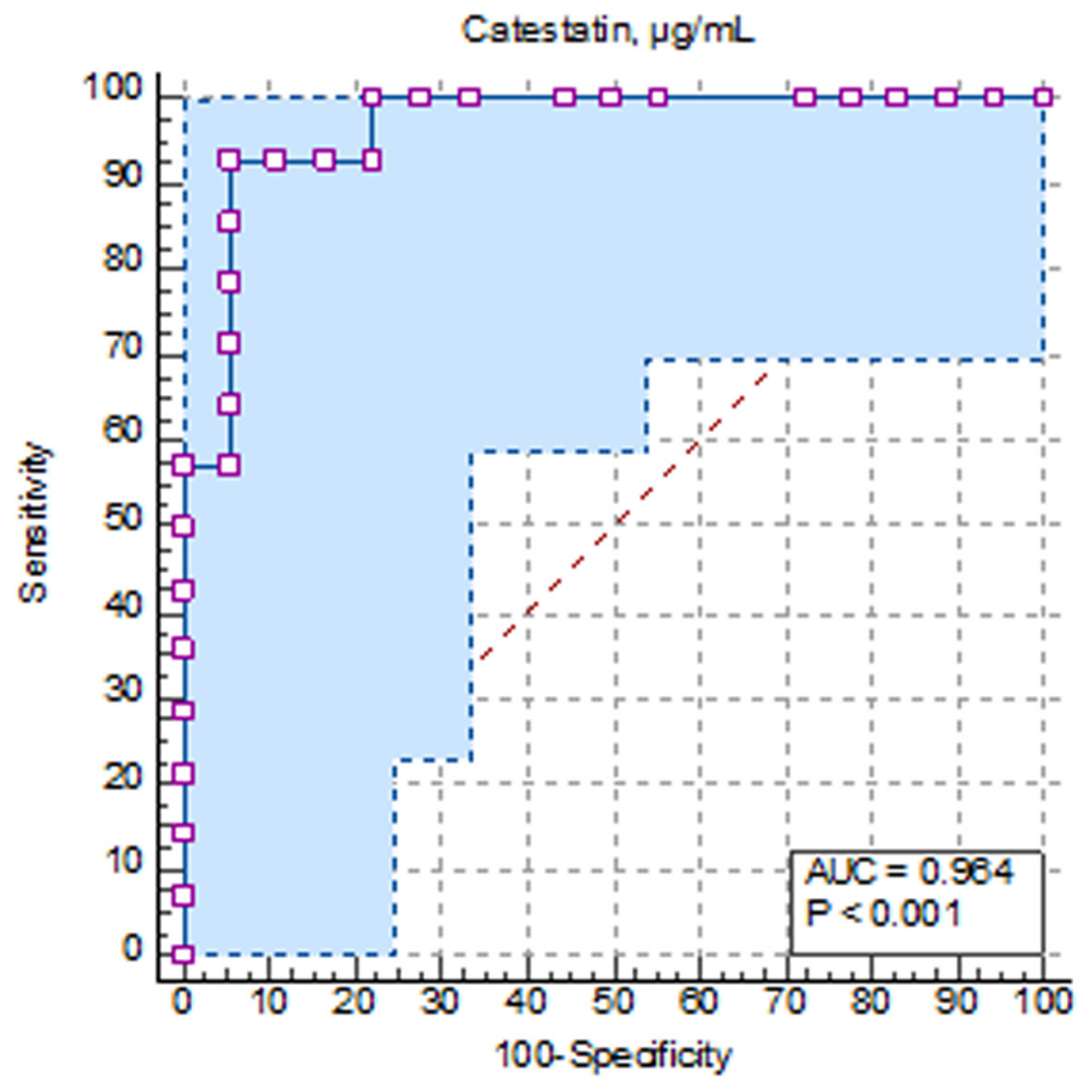
The ROC-curves of catestatin levels associated with adverse outcomes during 12 month follow-up period.

**Conclusion:** Decreased serum concentrations of copeptin and catestatin were associated with the presence of HFpEF and were related to overexpression of the biomarkers of fibrosis and inflammation in patients with non-obstructive CAD. Moreover, the serum values of copeptin were more linked with HRV parameters, when catestatin correlated with LV remodeling estimates. Catestatin may be used as a prognostic marker in HFpEF patients during 12 month follow-up period.

Abstract ID: 86

Track: Heart failure

## Supplemental support cardiac therapy-beyond the oxygen uptake rate

Mila Jakovljević

Polyclinic for Cardiovascular Diseases and Prevention Sveti Nikola, Korčula, Croatia

**Introduction:** Aim of the study was to assess the effects of supplemental support cardiac therapy/SSCT/on the heart function and quality of life.

**Methods:** Supplemental support cardiac therapy consists of a 15-day session and includes the use of coenzymes/substrates, oxygen, antioxidants, a low-frequency pulsed magnetic field and physical exercise (1,2). SSCT was applied in 106 sessions-30 sessions in patients with preserved EF and 76 sessions in patients with reduced EF. All patients underwent detailed M-mode ultrasound examination and 2D echocardiography and in 30 sessions EF and LV volumes were determined using 4D biplane volume measurement. In patients with preserved EF a cardiopulmonary echocardiographic test with physical exercise was performed at the start and the end of a 15-day session. At the start of the session, all patients received magnesium, niacin, Q-10 coenzyme, thiamine diphosphate, riboflavin, pantothenic acid, pyridoxal, biotin, glutathione and vitamin E. After physical stress the patients inhaled 95% oxygen, 4l/min through oxygen concentrator with ionisation while lying in a low-frequency pulsed magnetic field. After oxygen inhalation, the patients received carnitine, arginine, NADH, lipoic acid, selenium, and vitamin C. For patients with reduced EF, the SSCT did not include physical exercise. Individual medical therapy was optimised prior to each SSCT period. Statistical analyses were performed using SPSS Statistics version 17.0 and version 25.

**Results:** The values before SSCT compared to values after SSCT are in a strong and very strong correlation with the VAS, NYHA, LVIDd and EF indicators and in a very strong correlation with physical and emotional dimensions. The correlation coefficients for all 20 pairs of cardiopulmonary echocardiographic variables before and after SSCT range from 0.568 to 0.952. p values are less than 0.05 for all 20 pairs of cardiopulmonary echocardiographic variables.

**Conclusion:** SSCT, that means, supporting normal mechanisms for energy production, improves the cardiac function and quality of life in patients with both reduced and preserved EF.

Abstract ID: 1004

Track: Heart failure

## Effects of Daniellia oliveri leaf extract on cardiac antioxidants of rats treated with dimethylamine and Docking of constituents with selected cardiovascular proteins

Adeleke G. E*., Ayobami T. E., Adeyemi T., Adeleke F. O., Olajide R. O., Idowu C. T., Sulaimon L., Owoade O. A., Ayorinde I. O., Sijuade G. A., Fatuntele G. O., Shittu R. B., Kehinde A., Idepefo D. A., Balogun B., Peter B. O.

Department of Biochemistry, Faculty of Basic Medical Science, College of Health Sciences, Ladoke Akintola University of Technology, Ogbomoso, Oyo State, Nigeria.

**Background and Objective:** *Daniellia oliveri* plant is a folkloric medicine in African, and has been reported to be used in traditional medicine, and as an anti-inflammatory and antipyretic agent (Traore et al., 2021). Dimethylamine (DMA) is a known environmental toxicant. The heart is one of the organs in Beta-receptors are predominantly present, and these receptors are responsible for signaling in the sympathetic nervous system. When activated (agonized), the receptors triggers increase in heart rate, contractility and hypertension. On the other hands, when they are blocked, either by natural or synthetic agents, these physiologic processes are reversed (Basile et al., 2018). YES-Associated protein (Yap) is a transcriptional co-factor responsible for cardiomyocytes survival, and its up-regulation is implicated in pathological cardiac fibroblasts proliferation (Sharif-Sanjani et al., 2021). This study was therefore designed to investigate the phytochemical constituents of methanol leaf extract of the plant, and the effects on cardiac antioxidant status in male Wistar rats exposed to DMA. Furthermore, the study carried out some computational profiling of the major constituents of the extract on selected proteins implicated in cardiac activities.

**Methodologies:** *Daniellia oliveri* leaves were collected in July 2023 from Obamoro village, Iwo, Osun State, Nigeria. After air-drying and mechanical grinding, the powder (500 g) was subjected to Soxhlet extraction with methanol, to obtain *Daniellia oliveri* methanol leaf extract (DOMLE). The extract was analyzed using Atomic Absorption Spectroscopy (AAS) and High-Performance Liquid Chromatography (HPLC). Thirty-five male Wistar rats (Average of 154 g) were purchased, acclimatized and assigned into Seven (7) groups (A-G) with five rats per group. Group A served as positive control (given normal saline), while group B (negative control) was intraperitoneally injected with DMA (10 mg/kg) twice per week. Groups C – G were orally intubated with 50, 100, 150, 200 and 250 mg/kg of DOMLE, respectively every other day, together with DMA as stated. After 28 days, the rats were sacrificed by cervical dislocation, and the cardiac organ was excised and prepared into homogenate, which was used for antioxidant profiles, including Superoxide dismutase, catalase, glutathione –S-transferase, glutathione peroxidase and reduced glutathione following standard protocols. In-silico study was performed on rhamnetin (a major constituent of DOMLE from HPLC), using atenolol and metoprolol as standard beta -blockers, and verteporfin as YES-associated protein (Yap) using virtual screening Schrodinger.

**Results:** The AAS shows presence of calcium, magnesium, zinc, chromium, manganese, sodium, potassium, copper and iron in DOMLE (Table 1). The HPLC chromatogram (Figure 1) indicates presence of five major compounds, including alpha-caryophyllene (6.16%), L-borneol (10.89%), quercetin (41.48%), kaempferol (16.49%) and rhamnetin (14.65%). Antioxidant studies showed that DMA significantly elevated (p < 0.05) both SOD and catalase and reduced GPx, GST and GSH values relative to positive control. Co-treatment of DOMLE and DMA significantly lowered catalase, and elevated GST, GPx and GSH levels at all doses (Table 2). Docking study shows the binding affinities of the ligands with beta-adrenergic receptor as –5.90 kcal/mol (rhamnetin) against the standard beta-blockers, atenolol (–6.41 kcal/mol) and metoprolol (–6.21 kcal/mol) (Table 3). However, rhamnetin formed one hydrogen bond with Tyr427, and other bonds with Phe423 and Ser108 (Figure 2). Metroprolol formed one hydrogen bond with Tyr127 and other bonds with Cys202 (Figure 3), whereas atenolol formed no hydrogen bond, and other bonds with Asp131 and Phe419 (Figure 4). On docking with Yap, the binding affinities were found to be –4.92 kcal/mol (rhamnetin), against the standard drug verteporfin (–6.37 kcal/mol) (Table 3). Rhamnetin formed hydrogen bonds with Glu338 and Lys336, and other bonds with Leu389, Tyr312, Val308, Ala223, Met362 and Met358 (Figure 5), whereas verteporfin formed one bond with Thr339, and other bonds with Glu340, Glu338, Val337, Pro357, Cys359 and Lys336 (Figure 6).

**Table 1:** Elemental analyses of *Daniellia oliveri* methanol leaf extract using Atomic Absorption Spectroscopy.


MACRO ELEMENTS	CONCENTRATION (ppm)	TRACE ELEMENTS	CONCENTRATION (ppm)

Calcium	2.891 ± 0.12	Zinc	1.311 ± 0.02

Sodium	2.344 ± 0.03	Copper	0.057 ± 0.01

Magnesium	2.866 ± 0.25	Chromium	0.101 ± 0.03

Potassium	1.389 ± 0.41	Manganese	1.203 ± 0.05

–	–	Iron	0.168 ± 0.00


**Figure 1:**
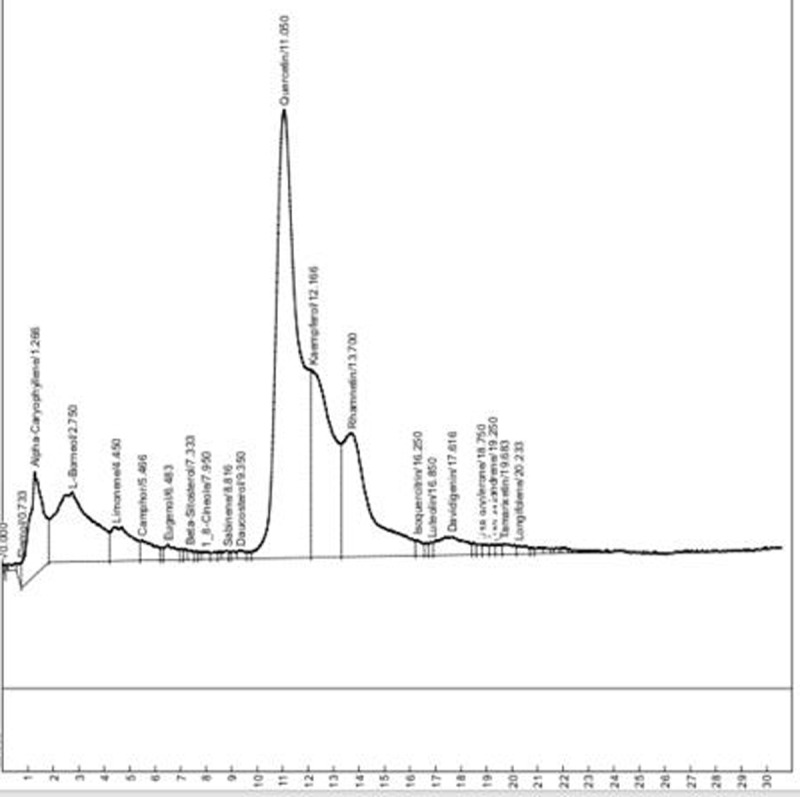
High-Performance Liquid Chromatography of *Daniellia oliveri* methanol leaf extract.

**Figure 2:**
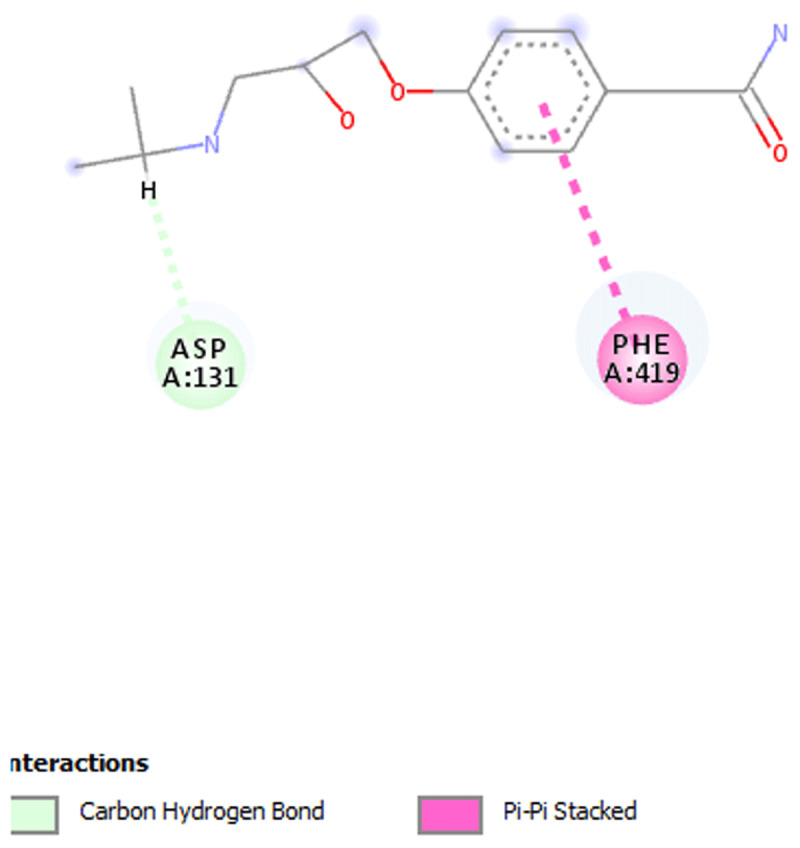
Molecular docking of Rhamnetin with.

**Figure 3:**
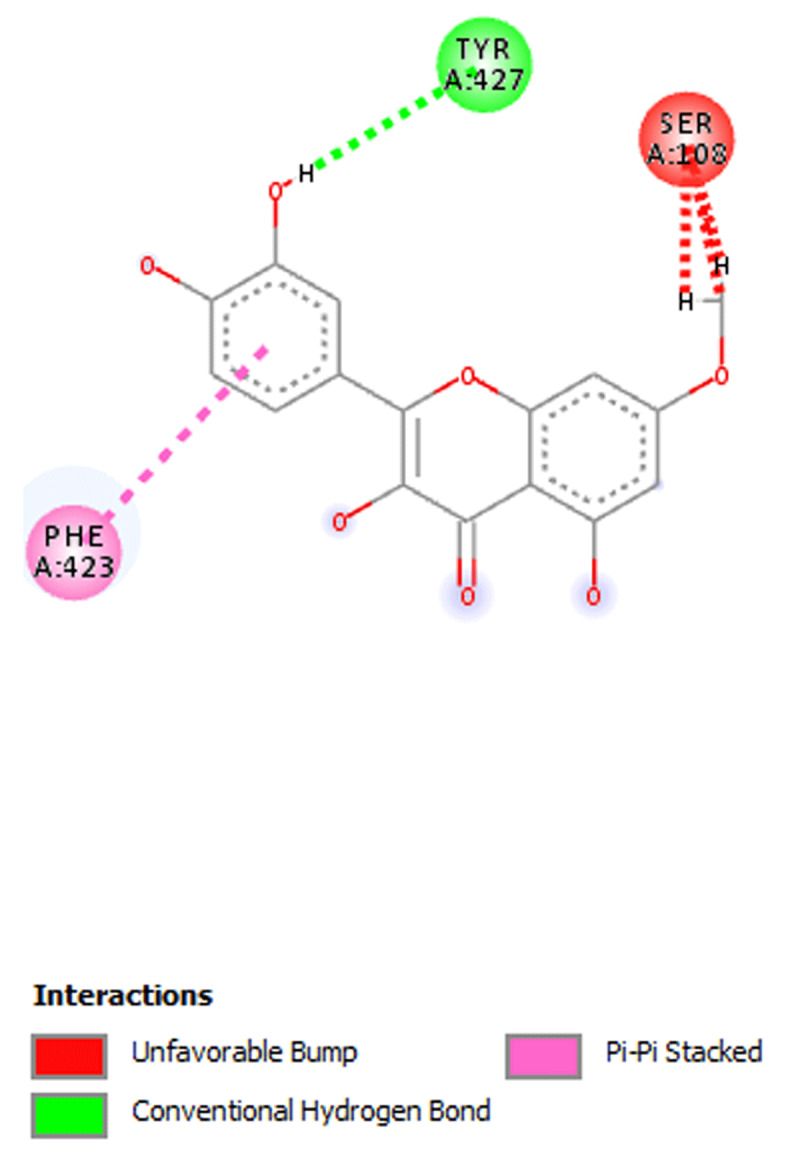
Molecular docking of Metroprolol with beta- adrenergic receptor beta-adrenergic receptor.

**Figure 4:**
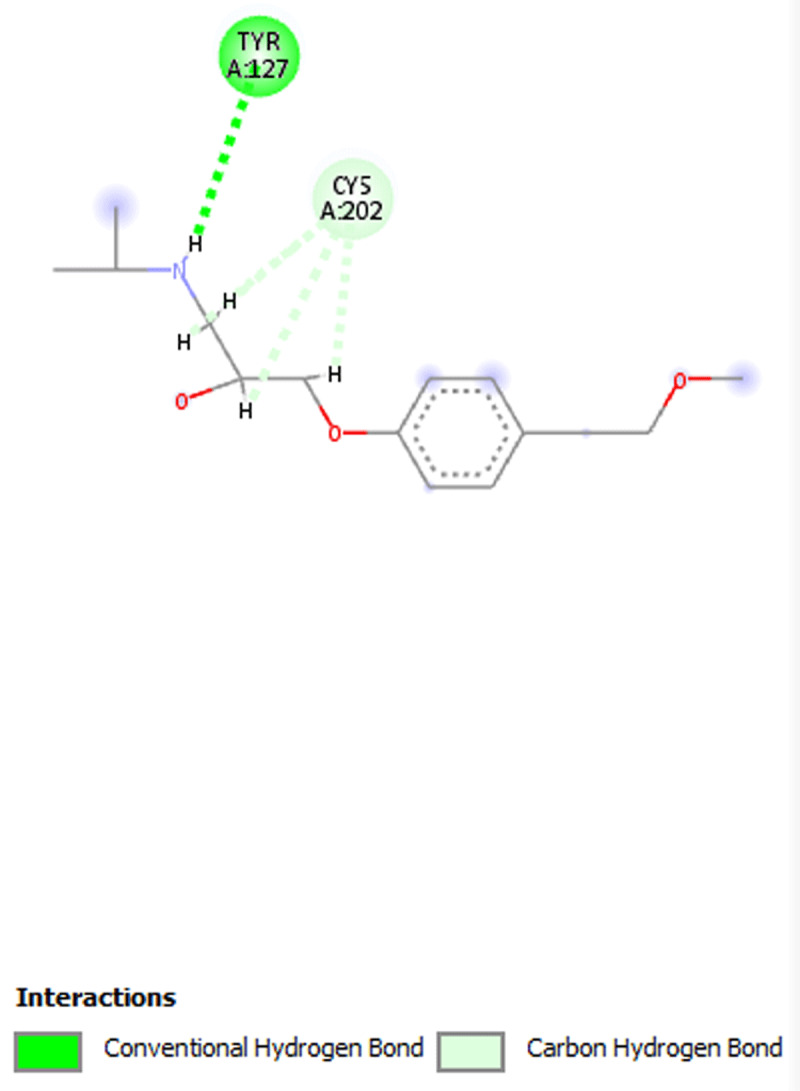
Molecular docking of Atenonol with beta-adrenergic receptor.

**Figure 5:**
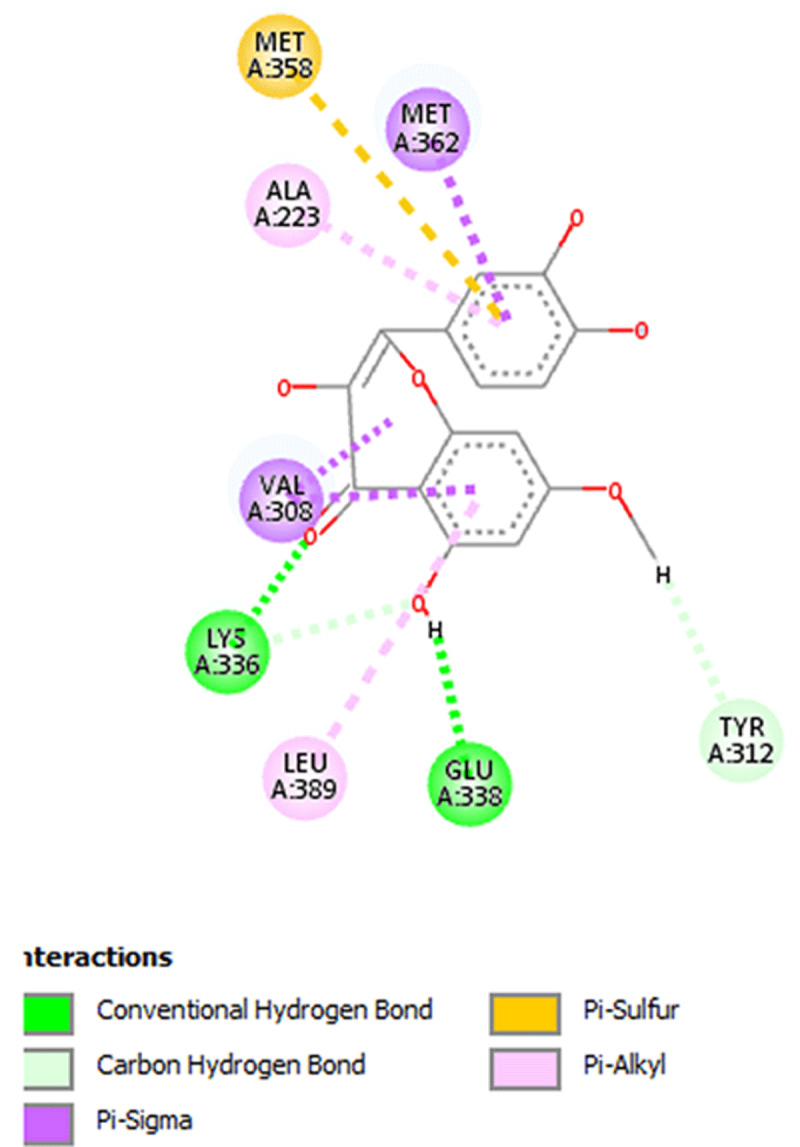
Molecular docking of Rhamnetin with Yap.

**Figure 6:**
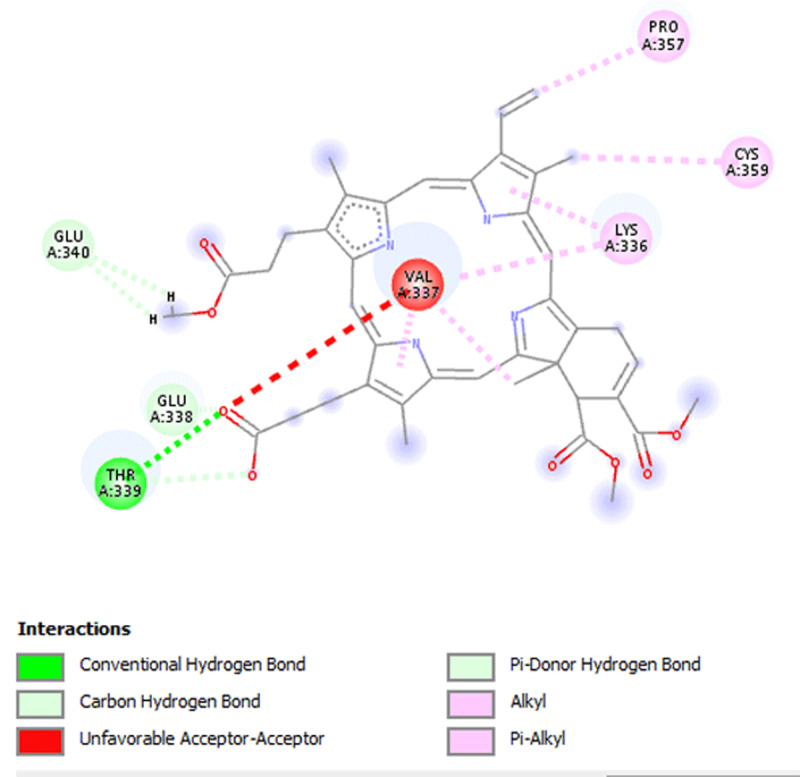
Molecular docking of Verteporfin with Yap.

**Conclusion:** This study has shown that *Daniellia oliveri* leaf extract is rich in physiologically significant minerals and phytochemicals. The phytochemicals could possibly potentially protect the cardiovascular system of rats via pathways involving antioxidants, beta-adrenergic receptor blocking and inhibition of YES-associated protein.

**Keywords:** antioxidant enzymes; cardiac system; computational study; *Daniellia oliveri*

Abstract ID: 325

Track: Hypertension

## Effect of Hydrolyzed Casein on Blood Pressure: A Systematic Review

Nicole Rodrigues Cardoso¹, João Vitor Freitas Bertuci^2^, Maria Fernanda de Miranda Rocha^3^, José Silva de Carvalho^4^, João Pedro do Valle Varela^5^, Sabrina Jorge Rodrigues^5^.

1-Santa Marcelina College, 2-Universidade Federal do Pampa, 3-Centro Universitário de Caratinga, 4-Universidade Cidade de São Paulo, 5-Faculdade Metropolitana São Carlos

**Background & Objective:** The initial treatment for high blood pressure involves non-pharmacological approaches, such as lifestyle and dietary changes, before considering medication. Food Peptides derived from hydrolyzed milk caseins, like Val-Pro-Pro (VPP) and Ile-Pro-Pro (IPP), are of interest alternative due to their potential to inhibit the angiotensin-converting enzyme, which could lead to lower blood pressure (1). This review aims to explore the impact of hydrolyzed casein on blood pressure reduction.

**Methodology:** A systematic review was conducted through the PUBMED, EMBASE, SciELO, and COCHRANE databases with the descriptors “Casein hydrolysate” and “blood pressure”. Eligibility, screening, and data extraction were performed by two independent reviewers.

**Results:** A total of 80 articles were identified, with 28 being duplicates. After screening titles and abstracts, 27 articles were excluded. The full text of 25 articles was reviewed, and 21 articles were excluded. These 4 articles included assessed blood pressure using standardized methods and compared the effects of hydrolyzed casein in yogurt or tablet form against placebos containing sodium caseinate. Regarding the dose of 6 mg of VPP and 7.6 mg of IPP, without lifestyle modifications (LM), there was a decrease of 10.5 ± 11.5 mmHg) for the intervention group and (–3.9 ± 9.6 mmHg) for the placebo group, and there was a significant difference (–6.63 mmHg, 95% [CI] –11.67 to –1.58, P < 0.05) (1). On average, after 4 weeks of treatment with 7.5 mg of VPP and 9.6 mg of IPP without LM, a reduction in SBP of 8.0–8.4 mmHg (p < 0.05) and DBP of 3.8–5.9 mmHg (p < 0.01) was observed (2). In the dose of 1 mg of IPP and 2 mg of VPP, the SBP in the intervention group without LM significantly reduced SBP compared to the start of the study (4.8 ± 8.7 mmHg, P = 0.013) (3). In the article comparing different doses of VLP and IPP and guiding LM, in the multivariate analysis, it was seen that the baseline SBP and the dose of the product were the only predictors of SBP reduction during treatment, as it was shown that the higher the Baseline SBP and the dose of the product, the greater the drop in pressure. Furthermore, it was seen that after 8 weeks, correcting the results with the placebo response, only the high-dose group (4.56 mg of IPP and 4.47 mg of VPP) showed a significant drop in DBP (p = 0.078), however, a dose-dependent effect on DBP reduction was shown (p = 0.05) (4).

**Conclusion:** Therefore, observing the data from these articles, it is concluded that hydrolyzed casein plays a significant role in reducing blood pressure with statistical significance. In this sense, there should be a promotion of more randomized clinical trials on the subject for a more reliable and up-to-date analysis of casein.

Abstract ID: 1006

Track: Hypertension

## Pooled, 12-month Blood Pressure Reductions Using the Symplicity Spyral Radiofrequency Renal Denervation Catheter

Tzung-Dau Wang^1^, Felix Mahfoud^2^, Raymond R Townsend^3^, Giuseppe Mancia^4^, David E Kandzari^5^, Robert Whitbourn^6^, and Michael Böhm^2^

^1^Department of Internal Medicine, National Taiwan University College of Medicine, Taipei, Taiwan, ^2^Universitätsklinikum des Saarlandes, Saarland University, Homburg, Germany, ^3^Perelman School of Medicine, University of Pennsylvania, Philadelphia, PA, USA, ^4^University of Milano-Bicocca, Milan, Italy, ^5^Piedmont Heart Institute, Atlanta, GA, USA, ^6^St Vincent’s Heart Centre, Melbourne, Australia

**Background and Objective:** Renal denervation (RDN) targets the sympathetic nervous system to lower blood pressure (BP). While BP reductions through short-term follow-up have been demonstrated, data on outcomes through longer term follow-up are limited. Here, we report the pooled, 12-month BP changes and safety outcomes from the largest existing RDN clinical program using the latest generation, multi-electrode, radiofrequency (RF) Symplicity Spyral™ catheter.

**Methodologies:** A broad spectrum of patients with hypertension were enrolled in the SPYRAL HTN Global Clinical Program, consisting of 4 major studies; SPYRAL First-In-Man, SPYRAL HTN-OFF MED, -ON MED and Global SYMPLICITY Registry (GSR) Denervation Findings in the Real World (DEFINE). GSR DEFINE was an all-comer registry for patients with uncontrolled hypertension, whereas patients enrolled in the SPYRAL HTN-OFF and -ON MED trials required to have office systolic BP (OSBP) ≥150 and <180 mmHg, office diastolic BP ≥90 mmHg, and 24-h ambulatory systolic BP ≥140 and <170 mmHg. All patients were treated with RF RDN using the Spyral catheter. OFF MED patients had a drug washout period prior to treatment and did not take antihypertensive drugs for the first 3 months. ON MED patients were on a stable 1–3 antihypertensive drug regimen at least 8 weeks prior to treatment and for 6 months after treatment. After primary endpoint ascertainment, patients were allowed to up-titrate drugs at the discretion of the physician. Reductions in office and 24-h ambulatory BP were pooled, and safety outcomes, including renal artery stenosis incidence (>70%, confirmed by angiography), were assessed.

**Results:** As of March 2023, 1,539 patients received RF RDN using the Spyral catheter (excluding crossover patients from sham control groups). Patients at baseline were 58 ± 12 yrs old, 38.2% female, 30.1% had type 2 diabetes, eGFR was 77.9 ± 24.4 mL/min/1.73 m^2^, 21.6% had chronic kidney disease stage 3 or more, OSBP was 164 ± 22 mmHg and 24-h ambulatory systolic BP (ASBP) was 152 ± 17 mmHg. The mean procedural time was 85.5 ± 46.2 minutes, the mean catheter time was 46.0 ± 25.2 minutes, the mean amount of contrast used was 156.3 ± 90.1 cc, and the mean number of ablations per patient was 39.2 ± 23.4 minutes. 12 months after RDN, OSBP changed by –15.9 ± 23.2 (n = 970; p < 0.001) and ASBP by –10.0 ± 15.7 (n = 660; p < 0.001; Figure 1). Significant diastolic BP reductions were also observed. There was no incidence of renal artery stenosis.

**Figure 1:**
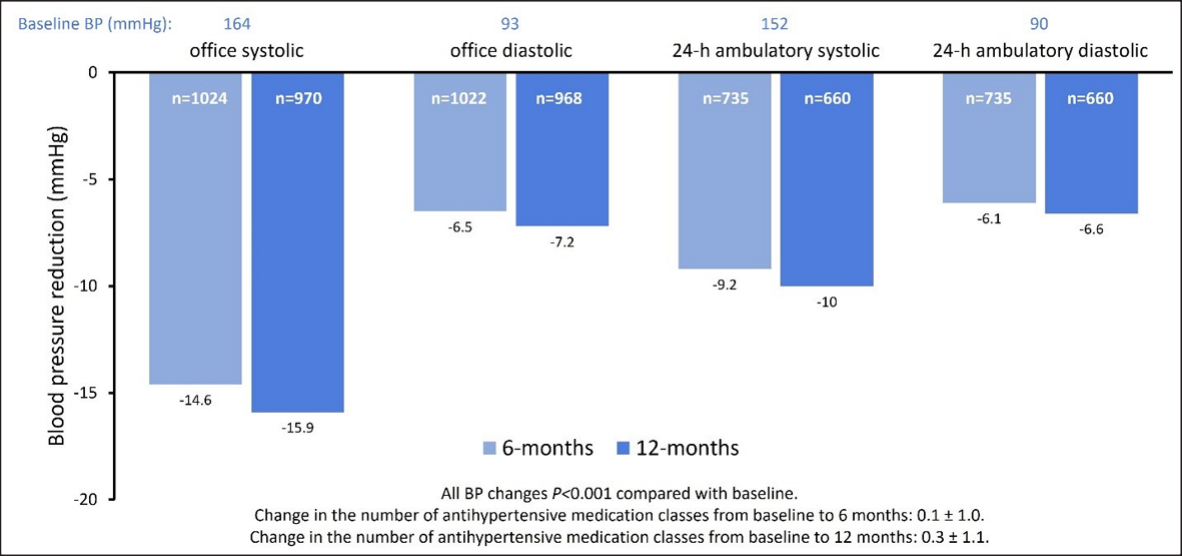
Comparison of blood pressure of patients receiving radiofrequency renal denervation (RF RDN).

**Conclusion:** In this large, pooled population of patients receiving RF RDN using the Spyral catheter, there were significant and consistent BP reductions through 12 months. There was no incidence of renal artery stenosis. These data highlight the safety and efficacy of RF RDN as an adjunctive therapy to antihypertensive drugs to treat high BP.

Abstract ID: 1099

Track: Hypertension

## Cost-Effectiveness of Radiofrequency Renal Denervation in Taiwan based on Contemporary Clinical Evidence

Tzung-Dau Wang, M.D., Ph.D., F.E.S.C.^1^; Khoa N. Cao, MBBS, MS, M.Sc.^2^; Anne M. Ryschon, MA^2^; Hui-Chun Huang M.D., Ph.D.^1^; Jan B. Pietzsch, Ph.D.^2^

^1^ Cardiovascular Center and Divisions of Cardiology and Hospital Medicine, Department of Internal Medicine, National Taiwan University Hospital, Taipei City, Taiwan, ^2^ Wing Tech Inc., Menlo Park, CA, USA

**Background & Objective:** Catheter-based radiofrequency renal denervation (RF RDN) is an interventional treatment for uncontrolled hypertension. In this study, the lifetime cost-effectiveness of RF RDN in the Taiwanese healthcare system was evaluated using latest available clinical evidence.

**Methods:** A decision-analytic Markov model previously published in the UK context was adapted to the Taiwanese health system setting. The model, based on multivariate risk equations that were adjusted to reflect event incidence observed in the Asian context, projected clinical events (stroke, myocardial infarction (MI), heart failure (HF), end-stage renal disease (ESRD), all-cause death (ACD)), quality-adjusted life years (QALYs) and costs over the patients’ lifetime. Risk reduction associated with changes in office systolic blood pressure (oSBP) in the treatment group was estimated based on 6-month data from the recent SPYRAL HTN-ON MED randomized, sham-controlled trial full cohort, including a base case effect size of –4.9 mmHg oSBP (observed vs. sham control). An alternative scenario considered an oSBP effect size of –29 mmHg as observed vs. baseline in the Taiwan subcohort of the Global Symplicity Registry (GSR). Costs in 2023 NT$ were based on published literature and procedural cost estimate. A 3.0% p.a. discount rate was applied for both costs and health effects. The incremental cost-effectiveness ratio (ICER) was evaluated against WHO-recommended thresholds of 1–3 times per-capita GDP (NT$ 1–3 million) per QALY gained. Extensive scenario and sensitivity analyses were performed.

**Results:** In the SPYRAL HTN-ON MED base case, RF RDN resulted in a reduction in clinical events (10-year RRs: Stroke = 0.80; MI = 0.88; HF = 0.74; ESRD = 0.95; ACD = 0.97) and an increase in health benefit over a patient’s lifetime, adding 0.25 QALYs at a concurrent cost increase of NT$216,381, yielding an ICER of NT$850,932 per QALY gained. The cohort characteristics and –29 mmHg effect size of the Taiwan GSR subcohort led to a projected lifetime QALY gain of 0.67 and decreased the ICER to NT$242,341 per QALY gained. Extensive scenario analyses demonstrated the robustness of cost-effectiveness findings, with probabilistic sensitivity analysis showing 76.3% and 100% likelihood of RF RDN being cost-effective at the NT$1 million and NT$3 million per QALY gained thresholds.

**Conclusion:** Model-based projections based on contemporary evidence suggest RF RDN leads to meaningful reductions in clinical events that render it a cost-effective intervention in the Taiwanese healthcare system for treatment of uncontrolled hypertension.

Funding support by Medtronic plc.

Abstract ID: 553

Track: Hypertension

## An Assessment Of Congenital Porto-Caval Shunts In Patients With Pulmonary Arterial Hypertension

Barkovskaia M., Kushnir V., Valieva Z., Martynyuk T.

National Medical Research Centre Of Cardiology named after academician E.I.Chazov of the Ministry of Health of the Russian Federation

**Introduction:** Currently congenital porto-caval shunts (CPCS) are considered among the causes of serious cardiopulmonary complications, including pulmonary arterial hypertension (PAH). Because the low prevalence of CPCS and the long asymptomatic period, it is important to describe clinical cases for early diagnosis of this malformation.

**Materials and methods:** We present an analysis of 6 clinical cases of patients with PAH (33% men), they received treatment in National Medical Research Center of cardiology named after academician E.I.Chazov of the Russian Federation from 2021 to 2023. All of them were diagnosed CPCS according to ultrasound results. The mean age of the patients was 47.3 ± 7.3 years. A duration of asymptomatic period, a frequency of symptoms and laboratory data were analyzed in all patients. Ultrasound was performed on the Voluson E-8 device. Also we used a convex transducer with a frequency of 3.5 MG in B-mode and a color doppler mapping (CDM) with ECG synchronization. The ultrasound results were compared with the results of right heart catheterization (RHC), contrast-enhanced computed tomography (CT).

**Results:** All of these patients had precapillary pulmonary hypertension by results of RCH. Such causes of PAH as congenital heart defects, pulmonary embolism, pulmonary diseases were excluded. The leading complaint was dyspnea at moderate physical load (climbing 1–2 floors). The average distance of the 6-minute walk test was 421.5 ± 51.2 meters, functional class 2 ± 0.3.

We founded on the ultrasound data and modern classification of CPCS and divided our patients into 2 groups: the first group – 3 observations (100% women), the portal vein was absent (Abernethy malformation type 1) and the second group – 3 cases (66.7% men), the portal vein was preserved (Abernethy malformation type II). There were mesenterico-caval shunts (2 observations) and mesenterico-renal shunt in first group. The second group was characterized by hypoplastic portal vein in 2 men and an open venous duct in a woman. All anomalies were founded by chance during the abdominal ultrasound during verifying genesis of PAH.

When analyzing these cases, we put forward a hypothesis that there is a correlation between right ventricular heart failure (RVHF) and anatomical proximity of the CPCS to the right atrium. Two patients (33.3%) had serious changes of systemic hemodynamics (mean pulmonary artery pressure by RCH 64 and 60 mmHg), visceral venous stasis and early clinical symptoms (5 and 15 years), also they were founded “high” CPCS relative the right atrium. During dynamic observation the patient with an open venous duct was noted a more rapid progression of circulatory failure which required a correction of therapy.

Four patients were diagnosed peripheral CPCS. They were multiple tortuous porto-caval junction between peripheral branches of superior mesenteric, splenic and left renal veins. These patients had changes in systemic hemodynamics (mean pulmonary artery pressure 57.8 ± 5.9 mmHg) without visceral venous stasis. The asymptomatic period averaged 44.5 years.

A differential diagnosis between congenital and acquired shunts was required for two patients with viral hepatitis. In favor of CPCS were the presence of hypoplastic portal vein, liver arterialization and enzyme values of reference.

**Conclusions:**

1. The analysis of these clinical cases show that a short asymptomatic period and the development of RVHF at a young age require the exclusion of CPCS.
2. Patients with “high” portocaval shunt had the most severe clinical situations.
3. Cases of complex comorbidity (CPCS + other liver diseases) require multidisciplinary approach.
4. The dependence of the clinical manifestation of CPCS and its anatomic proximity to the right atrium can be considered as a predictor of the development of severe RVHF and requires further studies.

Abstract ID: 1232

Track: Hypertension

## A Collaborative Model For Capacity Building Of Primary Care Physicians In The Management Of Hypertension And Diabetes In Mongolia

Oyundari T, Bujinlkham Sh, Suvd N, Enkh-Oyun Ts, Chantsaldulam P, Munkhchimeg G, Nasandelger G

Oddariya Foundation

**Background:** Non-communicable diseases such as arterial hypertension, type 2 Diabetes Mellitus (T2DM), and dyslipidemia is prevalent in Mongolia. The overall weighted prevalence of hypertension in Mongolia is 25.6% and hypercholesterolemia in the population was 61.9%. The high rates of such diseases are linked to inadequate education and awareness among the population, created by unhealthy lifestyles and behaviors within the population. Furthermore, these diseases are a serious burden on the patients, their families, and Mongolia’s healthcare systems due to a weak governance system and limited services.

**Objectives:** Reducing the burden on referral hospitals by providing optimal care at the level of family health centers for conditions like arterial hypertension, type 2 diabetes, and dyslipidemia involves implementing a comprehensive approach that focuses on prevention, early detection, and management.

**Methods:** Oddariya foundation and other professional associations jointly developed and delivered a countrywide capacity building program for primary care physicians in 2022–2024. Patient self-monitoring application was developed as an activity for patients with hypertension and diabetes. Through this application, the primary care physicians monitor their patient by physical and lab results of application. Before and after the start of the activities, doctors’ knowledge was assessed using a questionnaire developed from clinical guidelines. The monitoring of patients with hypertension and with diabetic under the supervision of doctors participating in the program was carried out through the application, and the improvement of laboratory parameters and the patient’s attitude were evaluated.

**Results:** A total of 122 primary care physicians participated in the capacity building interventions and 1757 doctors attended the training. Capacity-building interventions have improved the capacity of professionals to identify and manage risk factors for non-communicable diseases. The knowledge of diagnosis and treatment of arterial hypertension and diabetes increased in 29.9% among trained physicians. During the program period, HbA1c and lipid analysis parameters of 2119 patients were recorded and monitored by application. Mean HbA1c decreased from 5.8% in 2023 to 5.6% in 2024, with a trend toward significant improvement of 0.11% monthly (P < .001). Improvements in cholesterol control were not observed during the implementation period. However, the frequency of patients’ blood pressure measurement (n = 1064) and fasting glucose measurement (n = 822) increased. After the program, there was not significant changes in cases referred to referrals by above diagnosis.

**Conclusion:** Although the capacity building activities for primary care physicians can improve care for patients with hypertension and diabetes, it is crucial to scale-up interventions that allow for regular, systematic, and integrated care, especially at the lowest levels of care.

**Keywords:** Hypertension; Diabetes; Capacity Building; Primary Care Physicians

Abstract ID: 42

Track: Hypertension

## Association between neighbourhood socioeconomic inequality and paediatric hypertension

Xinjun Li

Center for Primary Health Care Research, Lund University, Sweden

**Objectives:** To examine whether there is an association between neighbourhood deprivation and incidence of childhood hypertension (CHP), after accounting for family- and individual-level sociodemographic characteristics.

**Methods:** All childhood born boys born in Sweden between January 1, 1979 and December 31, 1994 were followed from 1997 to 2017. The association between neighbourhood deprivation and the outcome was explored using Cox regression analysis, with hazard ratios (HRs) and 95% confidence intervals (95% CIs). All models were conducted in both boys and girls and adjusted for family- and individual-level variables and co-morbidities.

**Results:** During the study period, among a total of 668,299 boys and 633,517 girls, 10,573 (1.6%) and 11,443 (1.8%) were diagnosed with hypertension, respectively. Incidence of hospitalisation for CHP increased with increasing neighbourhood-level deprivation across all family- and individual-level sociodemographic categories. The hazards ratios (HRs) for CHP for those living in high-deprivation neighbourhoods versus those living in low-deprivation neighbourhoods were 1.22 (95% confidence interval (CI) = 1.14–1.30) in boys and 1.15 (95% confidence interval (CI) = 1.08–1.23) in girls. High neighbourhood deprivation remained significantly associated with higher HRs of CHP after adjustment for family- and individual-level sociodemographic characteristics (HR = 1.13, 95% CI = 1.06–1.22 in boys and HR = 1.10, 95% CI = 1.03–1.18 in girls).

**Conclusions:** This study is the largest so far on neighbourhood influences on CHP. Our results suggest that neighbourhood deprivation is associated with a moderately increased risk of CHP independently of family- and individual-level sociodemographic characteristics.

**Keywords:** Childhood hypertension; neighbourhood-level deprivation; incidence; sociodemographic factors

Abstract ID: 704

Track: Lifestyle, nutrition and primordial prevention

## The Contribution of Individual-Level Socioeconomic Factors, Health Conditions, and Health Behaviors to Cardiovascular Mortality: A United States Based National Sample

Adrian M. Bacong^1,2,3^, Malathi Srinivasan^2,4^, Latha Palaniappan^1,2,3^

1 Department of Medicine – Division of Cardiology, Stanford University School of Medicine, 2 Stanford Center for Asian Health Research and Education, 3 Stanford Cardiovascular Institute, 4 Department of Medicine – Division of Primary Care and Population Health

**Background:** Social determinants of health (SDH) are generally hypothesized to account for 20% to 40% of individual mortality (1,2). However, recent studies suggest that SDH alone may account for a much smaller mortality percentage (3). To date, no definitive formal studies quantify the exact mortality contribution of SDH in conjunction with other potential determinants.

**Methodology:** We use pooled 2007–2018 United States’ National Health and Nutrition Examination Survey data linked to the National Death Index through 2019 (4). Using Cox Proportional Hazard models with age as the time scale, we examined SDH (educational attainment, income-to-poverty ratio, annual familial income) contribution to cardiovascular mortality risk, accounting for demographics, standard modifiable cardiovascular risk factors (diabetes, hypertension, high cholesterol, smoking), health behaviors, biomarkers, and medication use. We then calculated the percentage variance explained by socioeconomic factors alone, then, alongside other covariates.

**Results:** Of socioeconomic factors, having a high school education (versus college education) and having less than 1.00 income-to-poverty ratio (versus 5.00+ income-to-poverty ratio) were independently associated with greater cardiovascular mortality risk (Table 1). Education alone explained 2% of cardiovascular mortality variance. Income-to-poverty ratio alone explained 1% of cardiovascular mortality variance (Table 2). Independently, all socioeconomic factors accounted for 2.4% of cardiovascular mortality variance. Incorporating all factors (except age) explained 19.5% of cardiovascular mortality variance. When age was included, total variance explained increased to nearly 50%.

**Table 2:** Multivariable Weighted Cox Proportional Hazard Models of Socioeconomic Factors and Covariates on Heart Disease Mortality, 2007–2018 NHANES, Unweighted N = 26,025.


VARIABLE	MODEL 1 INDEPENDENT CONTRIBUTION OF EACH FACTOR ON CARDIOVASCULAR MORTALITY			MODEL 2 ADJUSTMENTS TO MODEL 1 WITH DEMOGRAPHICS, STANDARD MODIFIABLE CARDIOVASCULAR RISK FACTORS*, HEALTH CONDITIONS, BIOMARKERS AND MEDICATION USE**		
	
	HR	95% CI	P-VALUE	HR	95% CI	P-VALUE

Educational Attainment						

College Graduate and	Ref.			Ref.		

Above						

<High School	1.56	1.05, 2.32	0.029	1.28	0.83, 1.95	0.257

High School Graduate	1.56	1.13, 2.16	0.008	1.42	0.99, 2.03	0.054

Some College	1.33	0.90, 1.94	0.148	1.22	0.83, 1.79	0.306

Missing	1.56	0.17, 13.99	0.689	1.13	0.12, 10.99	0.913

Income-to-Poverty Ratio						

5.00+	Ref.			Ref.		

<1.00	2.37	1.22, 4.60	0.011	2.37	1.16, 4.85	0.018

1.00–1.99	1.43	0.81, 2.55	0.217	1.47	0.79, 2.73	0.222

2.00–2.99	1.27	0.76, 2.13	0.348	1.22	0.71, 2.12	0.464

3.00–3.99	0.99	0.60, 1.65	0.970	0.98	0.59, 1.64	0.942

4.00–4.99	1.52	0.88, 2.61	0.132	1.48	0.85, 2.59	0.163

Missing	0.64	0.27, 1.48	0–290	0.64	0.26, 1.53	0.307

Annual Family Income						

$45,000+	Ref.			Ref.		

<$45,000	1.01	0.60, 1.70	0.959	1.05	0.61, 1.81	0.865

Missing	1.06	0.37, 3.03	0.915	1.21	0.40, 3.63	0.730


HR = Hazard Ratio; CI = Confidence Interval. Participant age is the time scale in all survival models. *Diabetes, hypertension, high cholesterol, smoking. **Anti-hypertensive, statin use.

**Table 3:** Explained Variance in Cardiovascular Disease Mortality.


DOMAIN	*%* VARIANCE EXPLAINED (95% CI)

Socioeconomic (SES) Factors	

Educational Attainment	2.27 (0.74, 4.58)

Income-to-Poverty Ratio	1.04 (0.08, 2.63)

Low Income Status	3.48 (-0.10, 1.53)

Education, Income-to-Poverty Ratio, Low-Income Status	2.41 (0.86, 5.09)

Covariates Alone	

Age Alone	28.52 (23.46, 33.76)

Sex and Race Alone	5.10 (2.59, 8.63)

Standard Modifiable Cardiovascular Risk Factors (SMURFs)Alone*	7.31 (4.40, 11.72)

Health Behaviors Alone**	4.68 (2.75, 8.26)

Health Conditions Alone***	5.0 (2.41, 8.95)

Biomarkers Alone^†^	8.06 (5.07, 12.95)

Medication Use^‡^	0.90 (0.41, 2.86)

Multivariable Models	

SES + Sex/Race	8.82 (5.83, 13.30)

SES + Sex/Race + SMURFs	13.75 (10.15, 19.27)

SES + Sex/Race + SMURFs + Health Behaviors	16.21 (12.87, 22.89)

SES + Sex/Race + SMURFS + Health Behaviors + Health Conditions + Biomarkers	19.32 (15.94, 27.20)

SES + Sex/Race + SMURFS + Health Behaviors + Health Conditions + Biomarkers + Medication Use	19.51 (15.93, 27.56)

SES + Sex/Race + SMURFS + Health Behaviors + Health Conditions + Biomarkers + Medication Use + Age	49.67 (44.68, 57.91)


*Hypertension, diabetes, high cholesterol, smoking. **Hours of sleep, alcohol use, physical activity, diet. ***Depressive symptoms, body mass index. ^†^Systolic blood pressure, total cholesterol, non-HDL-C cholesterol, eGFR, hemoglobin A1c. ^‡^Anti-hypertensive, statin use.

**Conclusions:** While individual-level socioeconomic factors alone account for only 2% of cardiovascular mortality variance, nearly 50% cardiovascular mortality variance is explained by the confluence of individual-level socioeconomic, demographic, health condition, and health behavior factors. Age is the largest mortality variance contributor. Future research should quantify additional individual-level contributors (e.g., genetic predisposition, stress, discrimination) and area-level factors (e.g., physical environment, food environment) that could explain the remaining 50% of variance. lives.

Abstract ID: 1173

Track: Multi-morbidity and metabolism

## The association of the number of chronic diseases with treatment burden among cardiometabolic patients in primary care in Hong Kong and mainland China

Zijun Xu^1^, Dexing Zhang^1^, Yang Zhao^2,3^, Samuel Yeung Shan Wong^1^

^1^JC School of Public Health and Primary Care, The Chinese University of Hong Kong, Hong Kong, China 999077, ^2^The George Institute for Global Health, University of New South Wales, Sydney, Australia 2050, ^3^The George Institute for Global Health China, Beijing, China 100600

**Background & Objective:** The prevalence of cardiovascular diseases is high in primary care patients, which received increased public health attention during the past several years. Cardiovascular diseases may act together with their complications and other diseases to increase the burden of disease and self-management. The purpose of this study was to evaluate the association of the number of chronic diseases with treatment burden in cardiometabolic patients in primary care.

**Methodologies:** In this cross-sectional study, self-report data were collected from multimorbidity patients with at least one cardiometabolic disease in three general outpatient clinics (GOPCs) in Hong Kong and six township health centers in rural areas or community health service centers in urban areas across three provinces in mainland China. Chronic diseases were measured using a checklist, which included 17 disease categories with 10 cardiometabolic diseases and 62 other common chronic diseases, which should be doctor-diagnosed and last for at least six months. Outcome measurement was the Patient Experience with Treatment and Self-management version 2.0 (PETS vs. 2.0) which measures treatment burden. The total score ranges from 0 to 100. Univariable and multivariable linear regressions were used to explore the association between the number of chronic diseases and treatment burden. The results were summarized as β and 95% confidence interval (CI).

**Results:** This study included 460 and 586 cardiometabolic patients in Hong Kong and mainland China. Among them, 32.2%, 30.0%, and 37.8% had 2, 3, and 4+ chronic diseases in Hong and that was 44.4%, 26.1%, and 29.5% in mainland China. In multivariable analysis, the increase in one disease was significantly associated with a 2.22-point increase in the overall treatment burden in Hong Kong (95% CI: 1.04, 3.40) and a 1.14-point increase in mainland China (95% CI: 0.04, 2.25) after adjusting for sociodemographic characteristics. Regarding different domains of treatment burden, the increase in the number of chronic diseases was associated with increased treatment burden in medical information, medication reliance bother, medication side effects bother, relationships with others, medical and healthcare expenses, difficulty with healthcare services, role and social activity limitations, and physical and mental exhaustion after adjustment (p < 0.05).

**Conclusion:** More chronic diseases were independently related to higher levels of overall treatment burden and its various domains. This highlights the importance of understanding and addressing the challenges faced by cardiometabolic patients in primary care to improve their overall well-being and quality of life.

**Keywords:** Cardiometabolic disease; Primary care; Treatment burden

Abstract ID: 1110

Track: Multi-morbidity and metabolism

## Patterns of comorbidities among in-hospital admissions from Pakistan: A Cardiovascular Disease Lens

Nida Saddaf Khan 1*, Mohummad Hassan Raza Raja 1*, Saad Bin Zafar Mahmood 2, Zainab Samad 1,2†

1 CITRIC Health Data Science Center, Aga Khan University, Karachi 74800, Pakistan, 2 Department of Medicine, Aga Khan University, Karachi 74800, Pakistan.

**Background & Objectives:** Rising life expectancies and improvements in medical care, have led to patients presenting to hospitals with an increasing number of comorbids. Comorbid conditions are also common in patients seeking cardiovascular care. This study aims to understand the patterns of comorbidities in a cohort of hospitalized patients from Pakistan.

**Methodology:** We included 161,015 adult patients (> 18 years), representing 234,225 admissions, between January 2011 and December 2021 admitted at a tertiary care hospital in Karachi. Comorbidities were classified as per the Charlson Comorbidity Index (CCI) and comorbidity clusters (groups with >1 comorbid) were formed on the basis of the 17 CCI variables, with the 10 most common CCI variables being chosen for further analysis. The comorbids and comorbidity clusters were represented graphically via an UpSet diagram and the trend for the top 10 comorbids was represented graphically via a line graph. All analysis was conducted via R and Python.

**Results:** Figure 1 displays the 40 most common comorbids and comorbidity clusters representing 144,564 (61.72%) admissions. Diabetes without complications was the most common comorbidity, present in 60499 (25.82%) admissions and included in 14/40 comorbidity clusters. The three clusters with the highest recorded admissions are:

1. Malignancy (except skin neoplasm) & metastatic solid tumor (7601 (3.25%))
2. Renal Disease & Diabetes with complications (5860 (2.50%))
3. Diabetes without complications & Myocardial Infarction (4081 (1.74%))

**Figure 1:**
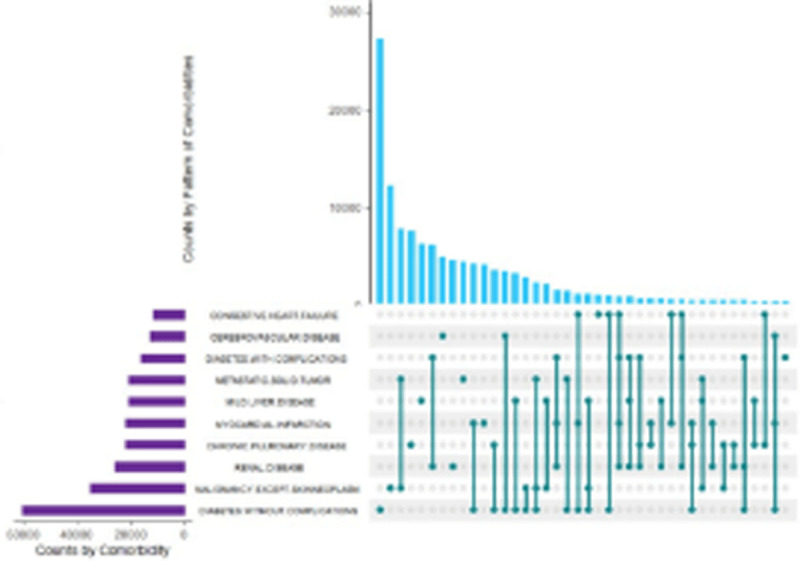
40 most common comorbids and comorbidity clusters.

Figure 2 displays a gradual rising trend of the top ten comorbids, with respect to the number of admissions (excluding the downtrend, thought to be attributable to the COVID-19 pandemic).

**Figure 2:**
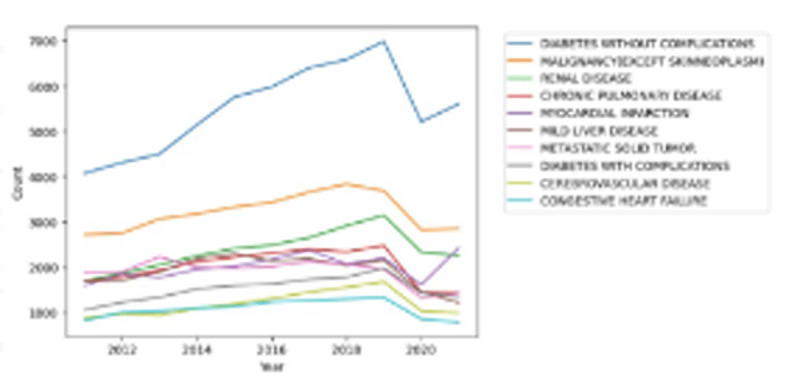
Trend of the top 10 comorbids (January 2011-December 2021).

Table 1 displays the top five comorbidity clusters with respect to the highest mortality rates. The cluster with the highest recorded mortality (68/447 (15.21%)) had Diabetes without chronic complications, Myocardial Infarction and Cerebrovascular Disease. Among the ten clusters with the highest mortality rates (15.21–7.37%), eight had cardiovascular disease.

**Table 1:** Comorbidity clusters with the highest mortality rates.


COMORBIDITY CLUSTER	CCI	ADMISSIONS (n)	GENDER MALE (%): FEMALE (%)	MEAN AGE (n ± SD)	MEAN LENGTH OF STAY (n ± SD)	MORTALITY (n (%))

Diabetes without Complications Myocardial Infarction Cereberovascular Disease	3	447	65.55: 34.45	66.65 ± 10.82	5.36 ± 5.56	68 (15.21)

Renal Disease Myocardial Infarction	3	523	70.17: 29.83	68.02 ± 13.65	5.32 ± 4.99	69 (13.19)

Malignancy (except Skin Neoplasm) Mild Liver Disease Metastatic Solid Tumor	7	527	58.82: 41.18	51.53 ± 13.57	4.58 ± 4.36	65 (12.33)

Myocardial Infarction Congestive Heart Failure	2	665	62.71: 37.29	68.49 ± 14.48	5.17 ± 4.76	68 (10.23)

Chronic Pulmonary Disease Myocardial Infarction	2	695	63.31: 36.69	69.54 ± 11.92	4.66 ± 4.62	69 (9.93)


CCI: Charlson Comorbidity Index.

**Conclusion:** There is a rising yearly trend in admissions with comorbidities. Cardiovascular disease (and its associated risk factors) continue to contribute significantly to morbidity and mortality of hospitalized patients, with eight out of ten clusters with the highest mortality rates, having cardiovascular disease.

Abstract ID: 674

Track: Other

## The Impact of Physician Age on Frailty Practices in Asia

Freda Cheng Yee Mah,^1^; Jie Jun Wong,^1^; Kay Woon Ho,^1,2^; Louis LY Teo,^1,2^; Jack Wei Chieh Tan,^1,2^; Junbo Ge,^3^; Angela S. Koh^1,2^

^1^ Department of Cardiology, National Heart Center Singapore, Singapore, ^2^ Duke-NUS Medical School, Singapore, ^3^ Department of Cardiology of Zhongshan Hospital, Fudan University, Shanghai China

**Background & Objective:** With populations aging around the world, rapidly mounting aging-related frailty, and cardiovascular disease (CVD) concurrent burdens are posing major threats to global population health (1). Particularly in Asian communities, the prevalence of frailty is alarmingly higher than the global frailty rate and represents substantial gaps in improving CV health, given the associations of unaddressed frailty with adverse CV outcomes (2). Despite the importance of system-based measures to recognize and tackle frailty, implementing specific frailty practices targeting frailty among older patients with CVD has yet to achieve its full potential and is still heavily reliant on opportunistic physician gestalt (1,3). We investigated the impact of physician age on frailty awareness, knowledge, and practices among cardiologists in Asian communities as key stakeholders in this advancing paradigm.

**Methodologies:** We launched a prospective multinational web-based survey via social networks to cardiology communities across China. Cardiologists were examined based on age, comparing under and above 50 years.

**Results:** Of 474 respondents (39.9% women), 65.0% were cardiologists below 50 years of age, and 35.0% were 50 and above, representing subspecialties including interventionists (48.5%), general cardiologists (32.3%), and heart failure specialists (9.3%). Most respondents practiced in the public healthcare setting (92.2%). Despite a smaller proportion of cardiologists aged ≥50 years, these older cardiologists were more likely to recognize the importance of frailty and more strongly believed that frailty screening and interventions would help reduce CV morbidity among their patients (definitely, 85.5% vs 66.3%, p < 0.001) compared to younger cardiologists. Older cardiologists were more confident in their ability to define frailty (confident/somewhat, 62.7% vs. 52.9%, p = 0.042), had greater awareness of the exercise modifications (well/vaguely aware, 74.7% vs. 65.2%, p = 0.035) that may benefit frail patients, and were also more likely to employ cardiac rehabilitation (frequently, 18.7% vs. 9.2%, p = 0.003), address polypharmacy (frequently, 53.6% vs. 35%, p < 0.001), and employ nutritional interventions (frequently, 23.5% vs. 9.8%, p < 0.001) or environmental modifications (frequently, 24.1% vs. 13.8%, p = 0.005), and engage in multidisciplinary team-based care (frequent 16.3% vs. 8.5%, p = 0.011) relative to their younger counterparts.

**Conclusion:** As key stakeholders, physicians have a frontline role in managing patients with frailty. Drawing on the knowledge, awareness, and strengths of older cardiologists to effect frailty strategies while addressing practice differences among the younger cardiologists may be necessary to implement frailty strategies.

Abstract ID: 373

Track: Rheumatic heart disease

## Comparison Between Annuloplasty and Mitral Valve Replacement in Rheumatic Heart Disease: A Systematic Review

Victoria Brandel Cruz^1^, Karolyna de Oliveira Ramos^2^, Diogo Barbosa Palhares^3^, Caio Cezar Ferreira Fraga^4^, Sabrina Jorge Rodrigues^5^, João Pedro do Valle Varela^5^

^1^ Universidade Federal de Juiz de Fora, ^2^ Universidade de Pernambuco, ^3^ Universidade de Franca, ^4^ Universidade Estadual de Feira de Santana, ^5^ Faculdade Metropolitana São Carlos

**Background & Objective:** Rheumatic disease is a group of medical conditions characterized by inflammation, pain, and limitation in movement. Cardiac involvement in rheumatic disease is one of the most serious manifestations of this condition, lead with heart valves, especially the mitral valve. Thus, cardiac involvement in rheumatic disease is a significant concern, because it leads to chronic cardiac complications, which require interventions, such as annuloplasty or even mechanical valve replacement. This review aimed to analyze the effects of mitral annuloplasty compared with replacement on the treatment of valvular complications in patients with rheumatic heart disease (1,2,3,4,5,6).

**Methodology:** This study employs a systematic review of the literature focusing on the descriptors “rheumatic heart disease”, “annuloplasty”, “mitral valve”, and “surgery”. The research was conducted by Health Sciences Descriptors (DeCS) and the strategy (((rheumatic heart disease)) AND (annuloplasty)) AND (mitral valve)) AND (surgery) was used, on the PUBMED, LILACS, Scielo, ScienceDirect and BMS databases. The PICO strategy was used to guide the search strategy that supported the data collection “In rheumatic heart disease, what is the effect of mitral annuloplasty on the treatment of valve complications compared with mitral replacement?”. The inclusion criteria were: articles published between 2014 and 2024; free; written in English, Spanish or Portuguese and that directly related the comprehensive understanding of interventions with the purpose of addressing mitral valve complications in rheumatic diseases.

**Results:** 57 articles were identified and, after the reading of the title and abstract, 17 articles were selected. Of these, 6 works met the eligibility criteria. It was identified that rheumatic fever (RF) had a higher prevalence in emerging countries, more specifically in the Pacific region, where RF is more prevalent in young children, aged 5 to 15 years, with Rheumatic Heart Disease (RHD) being the most common complication of RF. The articles that discussed the therapy of RHD pointed out the replacement of the mitral valve (MV) with a synthetic valve or the repair of the MV as possible treatment choices. The treatment approach for those affected must follow a systematic approach related to the patient’s profile and their cardiac condition, in particular the structural condition of the valve, in order to assess their eligibility for MV replacement or repair, such as in young and physically active patients, but with limited access to healthcare, for which the choice of mitral annuloplasty is given as a preference, as a temporary solution. In articles comparing the two therapies, it was observed that patients who underwent MV repair presented risks for reoperation, in addition to also presenting higher rates of mitral regurgitation compared to patients who underwent MV replacement with a mechanical valve. Regarding mortality from extracardiac causes, similar risks were found for both groups.

**Conclusion:** Mitral valve complications in rheumatic heart disease can be treated with repair or replacement of the valve. The choice between techniques depends on the patient’s profile. Annuloplasty was pointed out as preferably recommended, but at the same time presented greater risks for reoperation and higher rates of mitral regurgitation compared with MV replacement.

Abstract ID: 703

Track: Rheumatic heart disease

## Epidemiological Analysis of Intra-Hospital Mortality from Chronic Rheumatic Heart Disease in Children and Adolescents up to 19 Years of Age in the Five Regions of Brazil: A Decade of Study

Ana Carolina Silva Vieira¹, Clara Pereira Oliveira Tavares ^2^, Felipe José Silva e Silva^3^, Sabrina Jorge Rodrigues^4^, João Pedro do Valle Varela^4^

¹ Universidade Federal de Juiz de Fora campus GV, ^2^ Universidade Salvador (UNIFACS), ^3^ Universidade Federal do Pará, ^4^ Faculdade Metropolitana São Carlos

**Introduction and Objectives:** Rheumatic heart disease is chronic and causes damage to the heart valves after episodes of rheumatic fever (1). It is an autoimmune reaction resulting from streptococcal pharyngotonsillitis (2). Prevalent in middle- and low-income areas, it has a significant impact on children and young adults in Brazil, with carditis standing out as a critical clinical manifestation (3). This study aims to map the epidemiological profile of mortality from chronic rheumatic heart disease in children and adolescents in Brazilian regions.

**Methodology:** This is a quantitative, descriptive, retrospective and cross-sectional epidemiological study using data from DATASUS. We analyzed the notification of cases and deaths from Chronic Rheumatic Heart Disease in the five regions of Brazil over 10 years (2013–2022). Variables from the SUS Hospital Information System (SIH/SUS) were accessed with a residence filter, including total hospitalizations and deaths. We considered demographic data on race/color, gender and age. The mortality rate was calculated by dividing deaths by hospitalizations and multiplying by 100. Tables generated with Excel 2019. Use of public information, no ethical review required.

**Results:** During 2013–2022, Brazil recorded 4,464 hospitalizations for rheumatic heart disease, concentrated mainly in the Northeast and Southeast, accounting for almost 70% of hospitalizations. There was a higher incidence among men, blacks and extreme age groups (<1 year and 15–19 years) Table 1.

**Table 2:** Number of total hospitalizations, number of total deaths and mortality rate (%) due to Chronic Rheumatic Heart Disease, according to Brazilian regions, between 2013 and 2022.


REGION	HOSPITALIZATIONS	DEATHS	MORTALITY RATE (%)

Northern Region	418	24	5.74

Northeast Region	1,815	44	2.42

Southeast Region	1,247	36	2.89

Southern Region	321	19	5.92

Central-West Region	663	19	2.87

Total	4,464	142	3.18


The South region leads the way with 5.59%, predominantly affecting brown (14.81%) and black (14.29%) people, with a higher incidence in children under one year old (17.65%) and women (7.64%). The North had the second highest rate (5.74%), affecting the indigenous (25%) and white (13.64%) populations, especially the under one year age group (7.55%), followed by the 15–19 age group (6.67%), and most notably females (7.21%). In the Southeast, the rate is 2.89%, affecting more the yellow race (8.33%) and the 15 to 19 age group (4.77%), mainly males (3.32%). The Midwest comes next, with the fourth highest rate, affecting the black population (14.29%), especially children under one year old (6.74%) and fifteen to nineteen year olds (4.49%), with a predominance of males (3.43%). The Northeast had the lowest rate (3.64%), with the greatest impact on blacks (4.88%), children under one year old (4.44%) and predominantly males (2.47%).

**Conclusion:** Given the continental dimension of Brazil, it is speculated that cultural, socioeconomic and demographic differences between regions influence the incidence of streptococcal pharyngotonsillitis in these locations, just like rheumatic heart disease. Understanding this panorama is crucial for targeting investments in health policies and for viable prevention of the disease.

Abstract ID: 696

Track: Rheumatic heart disease

## Social vulnerability and the incidence of rheumatic fever in Brazil: a focus on the northeast

¹Julia Sander Santos, ¹Emily Amaral Gonçalves, ¹Milena Meiber Oliveira de Paula, ¹Sabrina Jorge Rodrigues, ¹João Pedro do Valle Varela

(1) Faculdade Metropolitana São Carlos

**Background & Objective:** Rheumatic fever is an inflammatory disease that can occur after infection with group A streptococci, commonly associated with untreated streptococcal pharyngitis. Despite being considered a controllable and preventable disease, rheumatic fever is still a public health problem in Brazil, especially in regions with high social vulnerability, such as the northeastern region of the country (1,3). With this in mind, the aim of this study is to expose the health vulnerability in which the population of northeastern Brazil lives, given that it is the region with the lowest Human Development Index (HDI) and with the most cases of rheumatic fever in the country, along with the highest number of deaths.

**Methodology:** This is a mixed systematic review, using numerical precepts and humanistic analysis to carry out the work (2). The data was collected through research platforms such as DataSUS, the Journal of the Brazilian Society of Cardiology and Scielo. The formulation and analysis of the data was verified by a decade of numerical analysis, between the years 2013 and 2023. The health descriptors and Boolean markers used to improve the research were the same (“Rheumatic Fever in Brazil” and “Social Vulnerability and Rheumatic Fever”). To improve the performance of this study, inclusion and exclusion criteria were created, adopting 2 of the 312 published qualitative studies and 1 table generated by DataSUS data.

**Results:** Analysis of the data between 2013 and 2023 revealed 21,524 cases of rheumatic fever in the country, followed by 581 deaths, representing a total of approximately 2,700 deaths per 100,000 inhabitants infected with the disease. In this environment, the northeast region of the country stands out, with 35.21% of the cases of infection and 36.7% of the cases of death, demonstrating the environment with the greatest risk of life for the inhabitants when it comes to rheumatic fever. However, the northeast of Brazil is a region known for its high social vulnerability, with health and development indicators below the national average. This vulnerability is reflected in the incidence of rheumatic fever, which is significantly higher in this region compared to other parts of the country. Poor living conditions, limited access to health services and a lack of basic sanitation are factors that contribute to the spread of streptococcal infection and, consequently, to the development of rheumatic fever.

In addition, the underreporting of rheumatic fever is a challenge faced in Brazil, especially in more vulnerable regions. Lack of awareness about the disease, difficulty in accessing health services and underestimation of symptoms can lead to under-reporting of cases, making it difficult to plan effective public policies to prevent and control the disease.

**Conclusion:** The incidence of rheumatic fever in Brazil is directly related to social vulnerability, with the Northeast being the region most affected due to its unfavorable socioeconomic conditions. Underreporting of the disease is an additional problem that compromises the effectiveness of public health actions. Therefore, it is essential that health policies are directed towards this region, with the aim of improving living conditions and promoting awareness of rheumatic fever, in order to reduce its incidence and mitigate its impacts on the most vulnerable population.

Abstract ID: 1174

Track: Structural heart disease

## Echocardiographic Assessment Of Mucopolysaccharidosis – Case Series

Devi Krishnan, Tamilselvan K., A. S. Arul, Justin Paul Gnanaraj, Nandha Kumaran, Manoharan G, Ashok Victor Pratap Kumar, Rajasekar Ramesh, Nageswaran

Institute of Cardiology, Madras Medical College and Rajiv Gandhi Government General Hospital, Chennai, Tamil Nadu, India

**Background:** Mucopolysaccharidosis are inherited lysosomal storage disorder caused by deficiency of enzymes, responsible for the degradation of glycosaminoglycans (GAG) and result in systemic deposition of GAG causing multisystemic manifestations. Cardiac findings, vary from asymptomatic valve involvement to severe heart failure (1,2). Echocardiography plays an important role in the assessment of cardiac manifestations in MPS patients, enabling early detection, monitoring disease progression, and guiding treatment decisions (3,4).

**Table:** 


SNO	AGE & SEX	MITRAL VALVE THICKNESS mm	AORTIC VALVE THICKNESS mm	MS MVOA cm^2^	MR	AS	AR	PHT	EF %	OTHERS

1	9 M	5.3	3.33	MILD, 2.6	MODERATE ECCENTRIC	NIL	MODERATE	NORMAL	68	PROMINENT TRABECULATION IN LV

2	13M	7.5	2.3	MILD 2.12	NIL	NIL	MILD	MODERATE	70	THICKENED TRICUSPID VALVE & SUB CHORDAL STRUCTUTRES, RVH, RVD

3	14M	10.3	4	SEVERE 1.5	MILD	NIL	MILD	MODERATE	62	THICKENED TRICUSPID VALVE, RVH

4	18 F	5.5	2.4	SEVERE 1.4	MILD	NIL	MODERATE	MODERATE	57	CHORDAL FUSION SEEN, THICKENED TRICUSPID VALVE

5	19M	5	3	MODERATE 1.7	MILD	NIL	MODERATE	NORMAL	64	

6	14	4.8	2.6	MODERATE	MILD	NIL	MILD	NORMAL	66	TRICUPSID VALVE THICKENED


**Methods and results:** In this case series we included 6 patients with a known diagnosis of MPS who visited our Cardiology OP. After collecting Demographic information, clinical features, and genetic analysis of these 5 patients, they were subjected to 2D/3D Echocardiography. using Phillips ECHO machine. The most frequently observed defect in this series are thickened atrioventricular valves and semilunar valves. Mitral stenosis was most the commonly noted, followed by aortic insufficiency and Mitral regurgitation. Arrhythmias were rare. Pulmonary hypertension was present in 4 cases. Coronary arteries were not involved.

**Figure 1:**
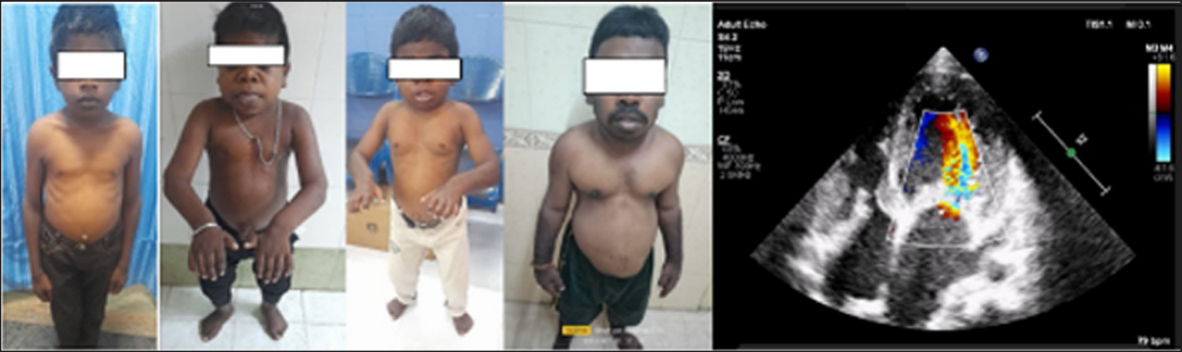
Photos of patients previously diagnosed with mucopolysaccharidosis with and an echocardiographic assessment.

**Discussion:** In MPS patients, accumulation of GAG in the endocardium and myocardium can cause cardiac involvement. Cardiac involvement is reported to be early and frequent in patients with MPS especially in types I, II and IV (1). Thickening and loss of function of the heart valve (especially in the left heart) and hypertrophy are common, and coronary artery involvement or conduction disorders are rare (2). The subvalvular apparatus of the mitral valve develops shortened chordae tendineae and thick papillary muscles resulting in dysmorphic and poorly mobile leaflets. The most common pathology reported was mitral valve insufficiency (51.4%), followed by aortic valve insufficiency (32.4%) where as in our series mitral stenosis was the commonly observed. Coronary artery narrowing and/or occlusion has been described in individuals with all types of MPS (3). Our cases does not have coronary involvement. LVH and diastolic dysfunction emerged at an early stage, whereas left ventricular dilation and systolic dysfunction occurred at older ages and later disease stage (4). They must undergo regular follow up and monitoring once in a year for further management.

**Conclusion:** This case series highlights the echocardiographic manifestation in MPS & its importance of early detection; as heart failure, arrythmia are the important cause of death. In our case series Mitral stenosis was commonly involved followed by aortic and tricupid regurgitations. Early diagnosis and appropriate treatment helps in saving life. Surgical intervention may be required in severe cases. Applicability of PTMC in these case is to be evaluated in future days. Further studies are needed to expand our knowledge in cardiac manifestation of MPS, and to guide appropriate management.
